# The Acute Effects of the Atypical Dissociative Hallucinogen Salvinorin A on Functional Connectivity in the Human Brain

**DOI:** 10.1038/s41598-020-73216-8

**Published:** 2020-10-02

**Authors:** Manoj K. Doss, Darrick G. May, Matthew W. Johnson, John M. Clifton, Sidnee L. Hedrick, Thomas E. Prisinzano, Roland R. Griffiths, Frederick S. Barrett

**Affiliations:** 1 Department of Psychiatry and Behavioral Sciences, Center for Psychedelic and Consciousness Research, Johns Hopkins University School of Medicine, 5510 Nathan Shock Drive, Baltimore, MD 21224 USA; 2 Department of Pharmaceutical Sciences, College of Pharmacy, University of Kentucky, Lexington, USA; 3 Department of Neuroscience, Johns Hopkins University School of Medicine, Baltimore, USA

**Keywords:** Neuroscience, Cognitive neuroscience, Computational neuroscience

## Abstract

Salvinorin A (SA) is a κ-opioid receptor agonist and atypical dissociative hallucinogen found in *Salvia divinorum*. Despite the resurgence of hallucinogen studies, the effects of κ-opioid agonists on human brain function are not well-understood. This placebo-controlled, within-subject study used functional magnetic resonance imaging for the first time to explore the effects of inhaled SA on strength, variability, and entropy of functional connectivity (static, dynamic, and entropic functional connectivity, respectively, or sFC, dFC, and eFC). SA tended to decrease within-network sFC but increase between-network sFC, with the most prominent effect being attenuation of the default mode network (DMN) during the first half of a 20-min scan (i.e., during peak effects). SA reduced brainwide dFC but increased brainwide eFC, though only the former effect survived multiple comparison corrections. Finally, using connectome-based classification, most models trained on dFC network interactions could accurately classify the first half of SA scans. In contrast, few models trained on within- or between-network sFC and eFC performed above chance. Notably, models trained on within-DMN sFC and eFC performed better than models trained on other network interactions. This pattern of SA effects on human brain function is strikingly similar to that of other hallucinogens, necessitating studies of direct comparisons.

## Introduction

Salvinorin A (SA) is a potent, selective κ-opioid receptor agonist and atypical dissociative hallucinogen found in *Salvia divinorum*, a plant only recently scheduled in many countries. Although *Salvia divinorum* leaves were traditionally administered orally by the indigenous Mazatecs of Mexico for ritualistic purposes^1^, the plant is now recreationally administered via vaporization or combustion. When inhaled, SA can produce intense feelings of depersonalization and derealization accompanied by drastic perceptual changes, with effects beginning within a minute of inhalation and subsiding by 15 min^2–4^. Similar to other hallucinogens like classic psychedelics (serotonin 2A or 5-HT_2A_ agonists like psilocybin, lysergic acid diethylamide or LSD, and *N*,*N*-dimethyltryptamine or DMT)^5–7^, dissociative anesthetics (NMDA antagonists like ketamine and dextromethorphan)^8–10^, and deliriants (muscarinic antagonists like scopolamine)^11–13^, some evidence suggests that SA may have rapid antidepressant effects^14,15^. Also like classic psychedelics^16,17^, other drugs with κ-opioid agonist activity such as ibogaine are being explored for their potential to treat addiction^18,19^, and SA, specifically, has shown efficacy in the treatment of preclinical models of cocaine abuse^20^. Unlike classic psychedelics, inhaled SA appears to be more incapacitating, causing unique alterations in interoception^4^, dense amnesia^3^, and more closely mimicking near-death experiences (similar to ketamine)^21^. One study reported SA experiences to be more similar to dreaming than experiences under other hallucinogens^22^ (cf. ^23^).

Recently, several human neuroimaging studies have investigated the acute effects of classic psychedelics and dissociative anesthetics, but only one of these, an electroencephalography study, investigated the effects of SA^24^. Like classic psychedelics^25–27^ and dissociative anesthetics^28,29^, SA decreased oscillatory power in low frequency bands at rest, suggesting that pharmacologically distinct hallucinogens have partially overlapping neural mechanisms. In functional magnetic resonance imaging (fMRI) studies, both classic psychedelics and dissociative anesthetics have been shown to decrease static functional connectivity (sFC; the strength of association between brain regions over time) within canonical resting state networks, especially within the default mode network (DMN)^25,30–35^, increase between-network sFC^25,31,35–37^, and increase or decrease sFC among visual regions^25,34,35,38,39^. A recent hypothesis suggests that classic psychedelics make the brain more “entropic,” particularly among interactions involving the DMN^40,41^. Several reports since have found classic psychedelics and dissociative anesthetics to increase various measures of variance and entropy across brain regions and functional connectivity patterns^42–45^, though the opposite has also been observed^25,46^.

The present study aimed to characterize the effects of inhaled SA on three measures of functional connectivity derived from fMRI data: static, dynamic (dFC), and entropic (eFC) functional connectivity. sFC was the standard measure of association strength between two regions’ timeseries (i.e., Pearson correlation), and dFC and eFC were defined as the variance and entropy, respectively, of sFC strengths over time. Whereas dFC can reflect large shifts between different connectivity strengths or states, eFC can reflect unpredictability in the distribution of different states of connectivity. In the following report, we tested the hypothesis that like other hallucinogens, SA would strongly modulate measures of DMN connectivity.

## Methods

### Participants

Twelve healthy male participants (23–52 years) were recruited through advertisements and word-of-mouth referrals (see Table 1 for demographics). One female participant completed the first SA dose but due to excessive movement and amnesia, was not continued. Screening included a phone and in-person psychiatric interview. Participants must have had ≥ 10 lifetime hallucinogen uses (e.g., 5-HT_2A_ psychedelic, NMDA dissociative, SA), 1 lifetime inhaled hallucinogen use (e.g., DMT, nitrous oxide, SA), 1 past year hallucinogen use, and 1 past year inhaled psychoactive drug use. Exclusion criteria included meeting DSM-V criteria for schizophrenia, psychotic disorder, bipolar I or II disorder, dissociative disorder, eating disorder, past two-year moderate or severe substance use disorder, or current major depression. Furthermore, individuals were excluded if they had a first or second degree relative with schizophrenia, psychotic disorder, or bipolar I or II disorder. Other exclusion criteria were pregnancy, nursing, current significant medical conditions, or standard fMRI contraindications (e.g., left-handed, incompatible medical devices). This study was approved by the Johns Hopkins Medicine Institutional Review Board, and all research was in accordance with the Common Rule and the Declaration of Helsinki. All participants provided informed consent.

**Table 1:** Demographic characteristics and drug use histories of study participants.

	Mean (SD) or percent
Age (years)	36.42 (8.10)
Education (years)	16.09 (2.12)
Weight (kg)	79.33 (13.78)
Percent White	92%
Percent Black	8%
Caffeine (cups/day)	1.05 (.98)
Nicotine (cigarettes/day in the five users)	.14 (.20)
Alcohol (drinks/week)	3.38 (3.02)
Cannabis (uses/month)	10.87 (14.23)
Lifetime uses of any classic psychedelics (5-HT_2A_ agonists, e.g., LSD, psilocybin)	251.33 (558.18)
Lifetime uses of inhaled classic psychedelics (e.g., DMT, 5-MeO-DMT)	7.75 (17.29)
Lifetime uses of entactogens (5-HT reuptake/releasing agent, e.g., MDMA, MDA)	29.25 (56.88)
Lifetime uses of dissociative hallucinogens (NMDA antagonists, e.g., ketamine, DXM)	12.53 (42.28)
Lifetime uses of Salvia divinorum	23.92 (50.40)
Years since last use of Salvia divinorum before medical screening in the 10 users	5.80 (5.11)

### Procedure

This study used a single-blind, placebo-controlled, within-subjects design. All participants completed an unblinded practice session and a single-blinded scanning session at Johns Hopkins University. Participants were asked to refrain from using psychoactive drugs 24 h before each session. At the beginning of each session, urine was drug tested, and negative results were required before proceeding. Prior to each session, participants were instructed to consume a low-fat breakfast and their usual amount of caffeine.

On the morning of the practice session, participants spent two hours with research personnel to build rapport and discuss the procedure. Participants were informed that they would receive a moderately high dose of SA. The inhalation procedure was practiced several times before drug administration. During these inhalations, participants laid in a supine position, wore eyeshades, and listened to music. Participants were prompted to exhale for five seconds while covering the end of the tube with their finger, as positive pressure could interfere with the vaporization process. During this exhalation, an experimenter began heating an empty flask with one butane micro torch. After the exhalation, participants were prompted to inhale through the tube for 45 s during which a researcher heated the flask with two torches. Participants were cued when there were 20, 10, 5, 4, 3, 2, and 1 s remaining after which they were prompted to exhale and drop the tube.

Once the inhalation procedure was sufficiently understood, a flask containing 15 µg/kg of SA was affixed to the delivery device (see Supplementary Information for description of drug and delivery device including analysis of tube deposition in Fig. S1). The inhalation procedure was then conducted during which an experimenter carefully moved the flames around the bottom of the flask and visually inspected that all SA was vaporized. Participants were then prompted by researchers to verbally rate the strength of subjective drug effects on a scale of 0 (no effect) to 10 (extreme, strongest imaginable) at 1, 2, 3, 4, 5, 10, 15, 20, 25, 30, and 45 min post-inhalation. If participants were unable respond, the rating was considered a 10. Prior to inhalation and 15 and 30 min post-inhalation, experimenters tested participants for tremors (see Supplementary Information for description of assessment). Other than these ratings, participants were encouraged to refrain from moving or talking until 30 min post-inhalation, and an experimenter kept a hand on participants’ shin to remind them of this. After 30 min, participants could remove their eyeshades and discuss their experience. After 45 min, participants completed several computerized questionnaires (see Supplementary Information for descriptions) and left the laboratory. Practice sessions were repeated for five participants due to excessive movement under SA or not completing the inhalation procedure properly.

The scanning session took place no more than one week after the practice session with nine participants completing both sessions on consecutive days. The scanning procedures consisted of an anatomical scan followed by two 20-min functional scans. Participants inhaled placebo (hot air) and 15 µg/kg of SA during the first and second scans, respectively. To minimize expectancy effects, participants were informed that one inhalation would be the dose of SA from their practice session, and the other inhalation could be a placebo or up to the dose of SA from their practice session.

Participants wore eyeshades and MR-safe headphones in the scanner. The inhalation procedure described above began approximately 45 s after the beginning of each functional scan. Recorded audio prompts for the inhalation were presented through the headphones, and after each inhalation, music played through the headphones for the remainder of the scan. No ratings were provided during scans. An experimenter remained in the scanner room with a hand on participants’ shin during functional scans. After scanning, participants completed questionnaires (see Supplementary Information for descriptions) and were debriefed.

### Analyses

**Figure 1:**
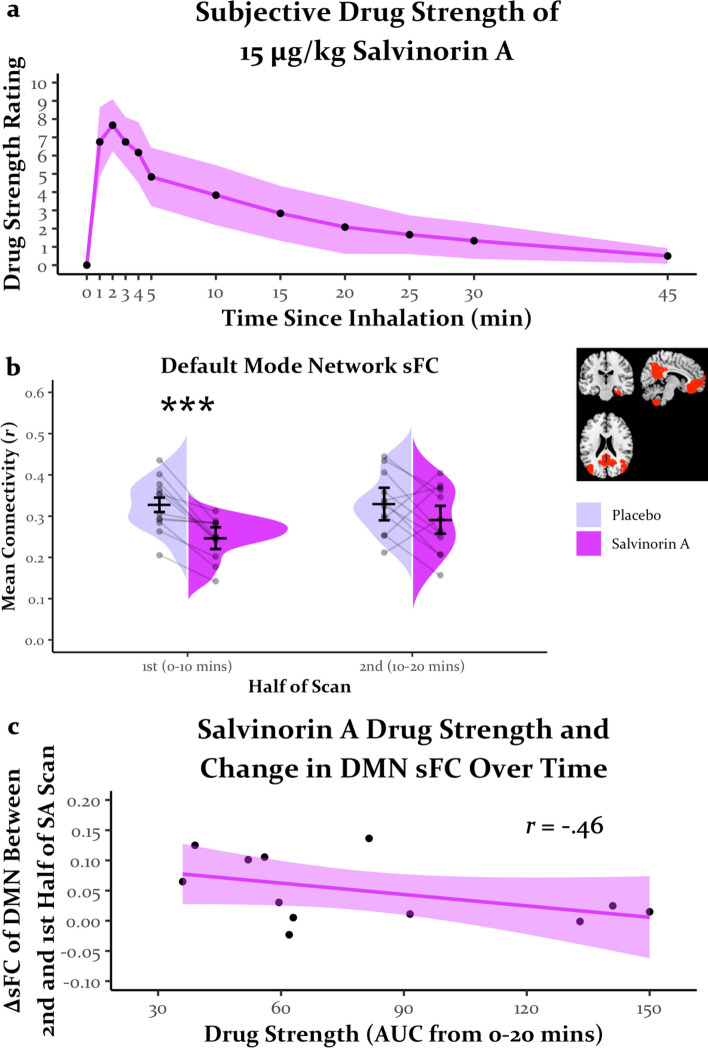
(**a**) Time course of subjective drug strength of salvinorin A as rated during the practice session. Ribbon around line reflects the 95% confidence interval. (**b**) Default mode network static functional connectivity as a function of drug condition and first or second half of each scan. Horizontal bars reflect the mean, and error bars reflect the 95% confidence interval. “Strings” on violin plots (lines connecting dots between drug conditions) reflect change in connectivity within individual participants and highlight the consistency of salvinorin A effects on reducing static functional connectivity in the first 10 min. ***p < .001. (**c**) Relationship between duration of subjective drug strength and change in default mode network static connectivity. Ribbon around line reflects the 95% confidence interval. Persistent attenuation of default mode network static connectivity by salvinorin A throughout the whole scan (measured as less change in connectivity between the second and first half of the salvinorin A scan) was moderately associated with longer duration of drug strength (greater area underneath curve for subjective drug strength ratings). sFC = static functional connectivity, DMN = default mode network, AUC = area underneath the curve.

Similar to previous reports^2,3^, the average subjective drug strength rating from the practice session dropped by approximately half from the peak rating by 10 min (i.e., equivalent in time to halfway through each scan; Fig. 1a). Therefore, after preprocessing (see Supplementary Information for preprocessing methods), the timeseries of all brain regions were split into first and second halves, creating a drug (placebo, SA) by time (first half of scan, second half of scan) design. See Supplementary Fig. S2 for split-half reliability analyses that further support such partitioning. Analyses repeated without partitioning timeseries did not change interpretation of results.

#### Static functional connectivity

Whole-brain sFC matrices were created for each participant and experimental condition by computing the Pearson’s *r* between the timeseries of all pairwise combinations of 268 Shen atlas^47^ regions (nodes), producing 35,778 functional connections (edges; see Supplementary Fig. S3 for single participant sFC matrices). All *r*-values were Fisher *z*-transformed for analysis. To complement our network-based analyses (see below), changes in all edge-wise static connections were also mapped. Edges were thresholded by one-sample *t*-tests across participants using a Bonferroni correction for all 35,778 edges. Although quite conservative, this procedure yields significant edges that are unlikely to represent noise in our small sample. Edges that survived thresholding for any condition were contrasted between drug conditions using paired *t*-tests (α = 0.05, uncorrected). This was the only analysis to apply this thresholding procedure.

#### Dynamic and entropic functional connectivity

Correlation timeseries were computed for each edge using dynamic conditional correlations (DCC)^48^. Compared to the commonly used sliding-window approach, DCCs do not suffer from artifacts introduced by arbitrary windowing practices, and dFC produced from DCC is far more reliable^49^. Whole-brain dFC and eFC matrices were computed by calculating the variance and approximation to differential entropy, respectively, of each correlation timeseries (see Supplementary Fig. S4 and S5 for single participant dFC and eFC matrices, respectively). The split-half reliability of all functional connectivity measures was good (see Supplementary Fig. S2).

In some prior fMRI work with hallucinogens^40,43^, entropy was analyzed at the level of the node. The present report analyzes the entropy of edges, as the focus is on functional connectivity. Furthermore, considering that the concept of information entropy is derived from information theory, information transfer is more intuitive at the level of the edge compared to the node. Because fMRI timeseries data are not integers, Shannon entropy cannot be calculated without discretizing the data. In one study^40^, a binning procedure was used to discretize data prior to calculating Shannon entropy, though the number of bins was not reported, nor were corrections made for the width of bins. Therefore, a histogram approximation to differential entropy was used:

$$ H\left( X \right) = - \,\mathop \sum \limits_{i = 1}^{n} f\left( {x_{i} } \right)\log \left( {\frac{{f\left( {x_{i} } \right)}}{{w\left( {x_{i} } \right)}}} \right) $$

where *f*(*x*_*i*_) is the discretized frequency distribution after binning and *w* is the bin width. Statistics resulting from a range of bin widths (15–120 bins, in steps of 15) did not meaningfully differ. Results are reported using 60 bins.

#### Network-based analyses

For each participant and experimental condition, network-based sFC, dFC, and eFC measures were computed by averaging all within-network edges for each of the 8 Shen atlas networks (medial frontal, frontoparietal, default mode, subcortical-cerebellum, somatosensory-motor, medial visual, occipital pole, and lateral visual) and all between-network edges for each of the 28 between-network pairings. *T*-values from contrasting drug conditions across participants were plotted in matrices to visualize within- and between-network changes.

#### Connectome-based classification

Given the small sample, connectome-based classification (e.g., similar to connectome-based predictive modeling^50–52^) was used to test the internal validity of the data. This technique identifies the connectivity measures (sFC, dFC, eFC) and network interactions most predictive of drug effects. To this end, a partial least squares (discriminant analysis) model was trained using a “leave-two-participant-out” cross-validation. At each iteration, a model was trained on 10 participants, and data from the remaining two participants in each condition (i.e., placebo first half, placebo second half, SA first half, SA second half) were classified as one of the four conditions. This procedure was repeated by leaving out all possible combinations of two participants (66 training/testing cycles) to create confusion matrices. This analysis was first performed using full connectomes of sFC, dFC, and eFC alone and in all combinations. Then, to determine whether within- or between-network connections were particularly predictive of SA effects on brain function, the classification procedure was repeated by training models on within- or between-network connectomes. Discrimination scores (*d*’) for each connectome were then computed from the proportion of data correctly identified as SA first half and the proportion of data mislabeled as SA first half. Given the enormous search space, models were not trained on combinations of within- and between-network connectomes.

## Results

### Subjective drug effects

Inhaled SA had its expected effects on the time course of drug strength ratings during the practice session similar to previous reports^2–4^, peaking 1–2 min post-inhalation, decreasing by approximately 50% at 10 min, and largely subsiding by 15–20 min, though the decay was somewhat variable (Fig. 1a). See Supplementary Table S1 for retrospective subjective measures.

### Static functional connectivity

**Figure 2:**
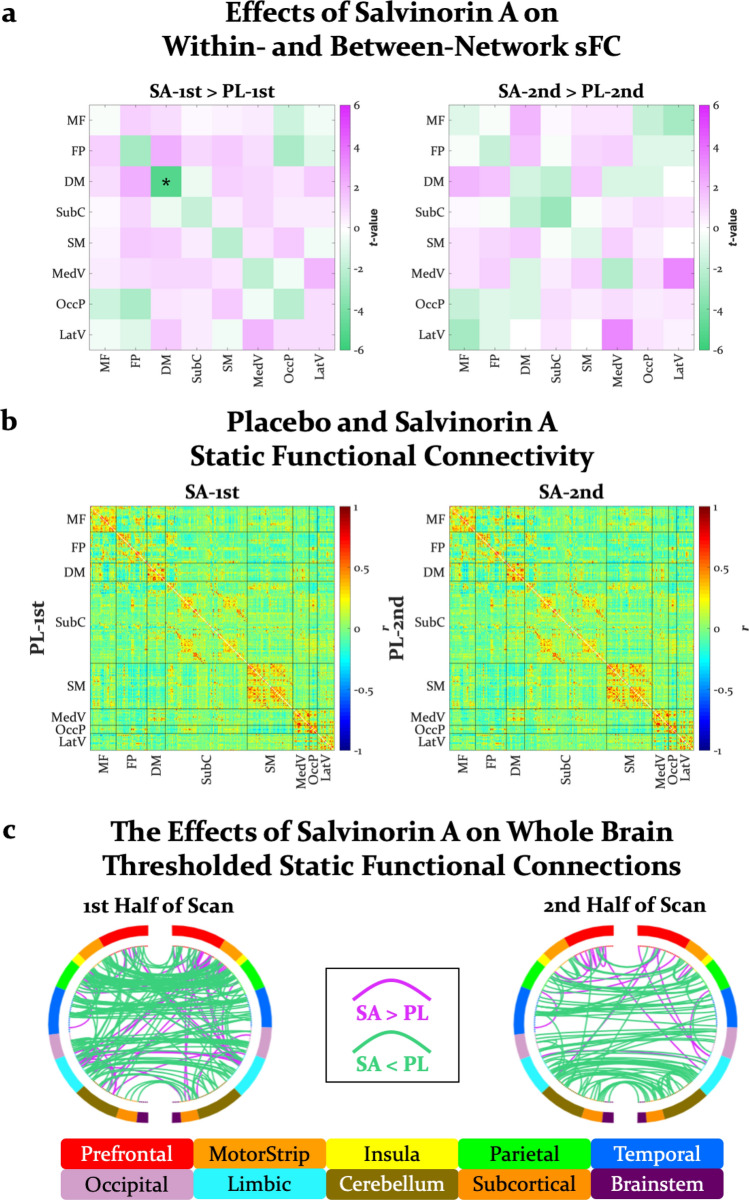
(**a**) Differences (*t*-values) in static functional connectivity within- and between-networks for salvinorin A vs. placebo in the first and second half of scans. Each row and column represent a single brain network as defined by the Shen functional brain atlas. The diagonal and off-diagonal cells represent differences in within- and between-network connectivity, respectively. (**b**) Static functional connectivity matrices for all pairwise functional connections (268 nodes, 35,778 edges) from the first and second half of placebo and salvinorin A scans, averaged across participants. Each row and each column represent a single node as defined by the Shen functional brain atlas, and the lower and upper triangles represent placebo and salvinorin A scans, respectively. The color of each off-diagonal cell represents the Pearson correlation (*r*) for an edge’s static connectivity. Nodes are grouped together in rows and columns by network as defined in the Shen atlas, with black lines marking the border between networks in the matrix. (**c**) Whole-brain static functional connections thresholded (α = .05, Bonferroni corrected for 35,778 edges) and contrasted between salvinorin A and placebo conditions (α = .05, uncorrected) for the first and second half of scans. As would be expected from (**b**), the connections that tended to survive were within-network (specifically, bilateral connections between homologous regions). SA = salvinorin A, PL = placebo, sFC = static functional connectivity, MF = medial frontal network, FP = frontoparietal network, DM = default mode network, SubC = subcortical-cerebellum network (includes the salience network), SM = somatosensory-motor network, MedV = medial visual network, OccP = occipital pole network, and LatV = lateral visual network. *p < .05, Holm-Bonferroni corrected for all 36 within- and between-network comparisons. Connectivity visualization from BioImageSuite (https://bioimagesuiteweb.github.io/webapp/connviewer.html).

Similar to previous investigations with classic psychedelics^25^, SA numerically decreased within-network (in 7 of 8 networks) but numerically increased between-network (in 21 of 28 network pairs) sFC in the first half of the scan (Fig. 2a). Consistent with other hallucinogens^25,30–33^, SA had its most robust attenuations on DMN sFC (Fig. 1b; *F*(1, 11) = 9.40, *p* = 0.011, ${\eta }_{P}^{2}$ = 0.46), particularly during the first half of the scan. Although a drug by time interaction was only trending (*F*(1, 11) = 3.50, *p* = 0.088, ${\eta }_{P}^{2}$ = 0.24), every participant exhibited reductions in DMN sFC in the first half of the SA scan compared to the first half of the placebo scan (95% CI: [0.05, 0.13], *t*(11) = 5.183, *p* < 0.001, *d* = 1.50). In contrast, this reduction was much more variable in the second half of scans (95% CI: [-0.03, 0.11], *t*(11) = 1.38, *p* = 0.195). Results from a Bayesian ANOVA provided practically no evidence for a model without an interaction term over the full model (*BF* = 1.07). No other changes in sFC survived multiple comparison corrections (see Supplementary Table S2 for all drug by time ANOVAs), perhaps due to the rapid decay of SA effects.

Due to the variability of SA effects on DMN sFC during the second half of the scan and in the decay of subjective drug strength from the practice session, a comparison between these drug effects was conducted. A moderate but nonsignificant relationship (*r* = − 0.46, *p* = 0.132) was observed between the change in DMN sFC from the first to second half of the SA scan and the area underneath the curve (AUC) of drug strength ratings for the first 20 min (i.e., the duration of scans; Fig. 1c). Those participants whose DMN remained less connected under SA in the second half of the scan were also those who experienced longer lasting subjective effects. Although AUC of drug strength could reflect peak magnitude, the magnitude and timing of peak effects was consistent across participants, suggesting that individual differences in AUC reflect duration of drug strength.

Because the strongest sFC edges were predominantly within-network connections regardless of experimental condition (Fig. 2b), the thresholding procedure disproportionately produced within-network connections, specifically bilateral connections between homologous regions (Fig. 2c). Fewer static connections under SA compared to placebo survived thresholding (first half of SA and placebo scans, respectively: 350 vs. 514; second half of SA and placebo scans, respectively: 405 vs. 457), and more of these edges were significantly attenuated under SA compared to placebo (first half: 161 vs. 40, second half: 96 vs. 16).

### Dynamic and entropic functional connectivity

**Figure 3:**
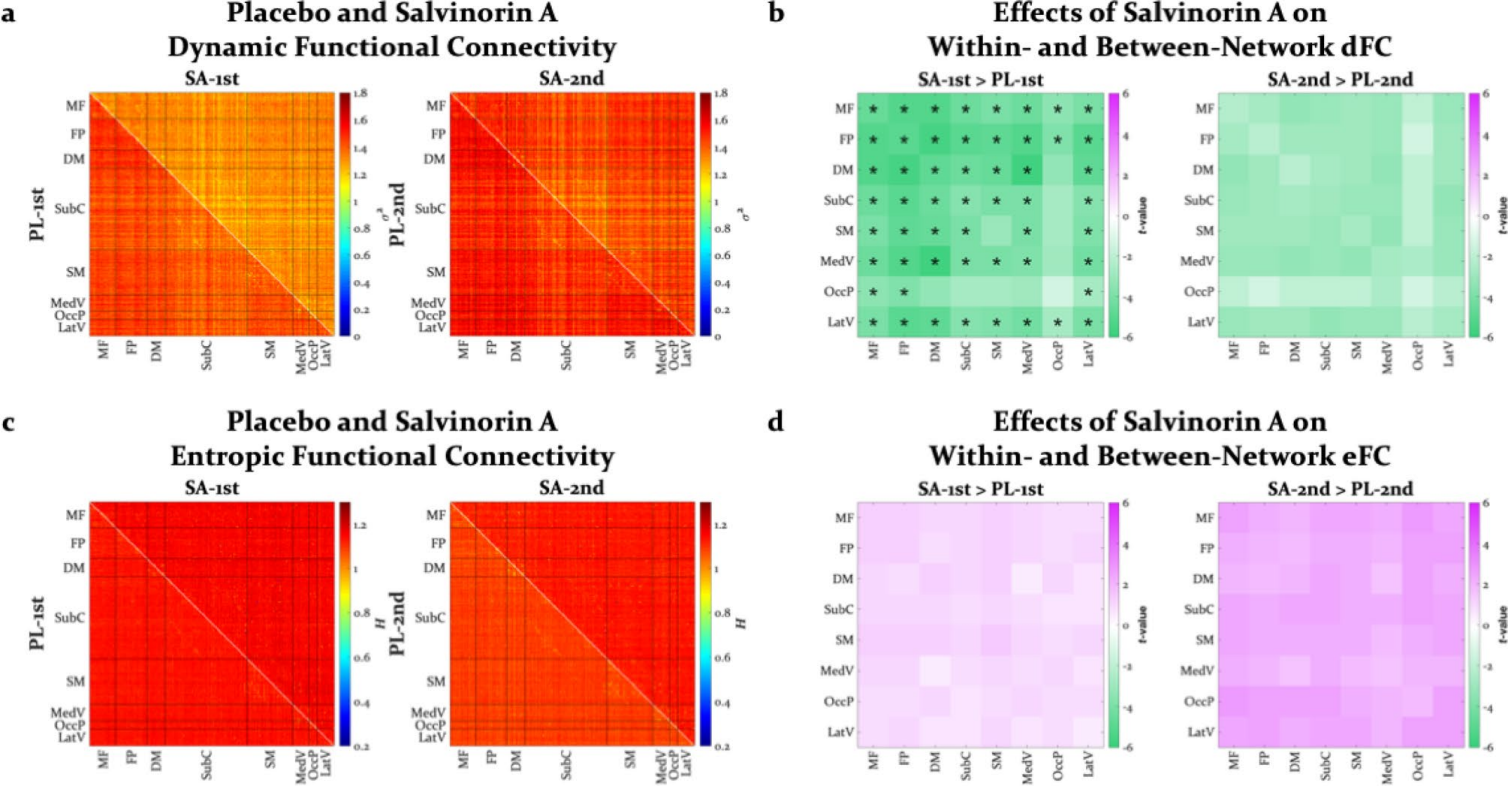
(**a**) Dynamic and (**c**) entropic functional connectivity matrices for all pairwise functional connections (268 nodes, 35,778 edges) from the first and second half of placebo and salvinorin A scans, averaged across participants. Each row and each column represent a single node as defined by the Shen functional brain atlas, and the lower and upper triangles represent placebo and salvinorin A scans, respectively. The color of each off-diagonal cell represents the variance (σ^2^) and entropy (*H*) of the correlation timeseries for an edge’s dynamic and entropic connectivity, respectively. Nodes are grouped together in rows and columns by network as defined in the Shen atlas, with black lines marking the border between networks in the matrix. Differences (*t*-values) in (**b**) dynamic and (**d**) entropic functional connectivity within- and between-networks for salvinorin A vs. placebo in the first and second half of scans. Each row and column represent a single brain network as defined by the Shen functional brain atlas. The diagonal and off-diagonal cells represent differences in within- and between-network connectivity, respectively. SA = salvinorin A, PL = placebo, MF = medial frontal network, FP = frontoparietal network, DM = default mode network, SubC = subcortical-cerebellum network (includes the salience network), SM = somatosensory-motor network, MedV = medial visual network, OccP = occipital pole network, and LatV = lateral visual network, dFC = dynamic functional connectivity, eFC = entropic functional connectivity. *p < .05, Holm-Bonferroni corrected for all 36 within- and between-network comparisons.

In contrast to sFC matrices, visual inspection of dFC and eFC matrices revealed widespread reductions in dFC but widespread increases in eFC under SA (Fig. 3a,c). Similarly, SA numerically reduced dFC and increased eFC within and between all networks (Fig. 3b,d), but only decreases in dFC during the first half of the scan survived corrections for multiple comparisons. Drug by time interactions for within- and between-network changes in dFC were also significant (see Supplementary Table S2 for all drug by time ANOVAs). When running these analyses without aggressively correcting for motion, individual differences in the degree of motion within a scan (i.e., framewise displacement) were correlated with individual differences in dFC and eFC for some within- and between-network cases. However, with more rigorous motion reduction (i.e., the data displayed here), these correlations were reduced, and the change in dFC became stronger (see Supplementary Fig. S6–S11 for motion-related analyses). Moreover, changes in eFC were negatively correlated with motion, suggesting that motion may have actually attenuated these effects.

### Connectome-based classification

**Figure 4:**
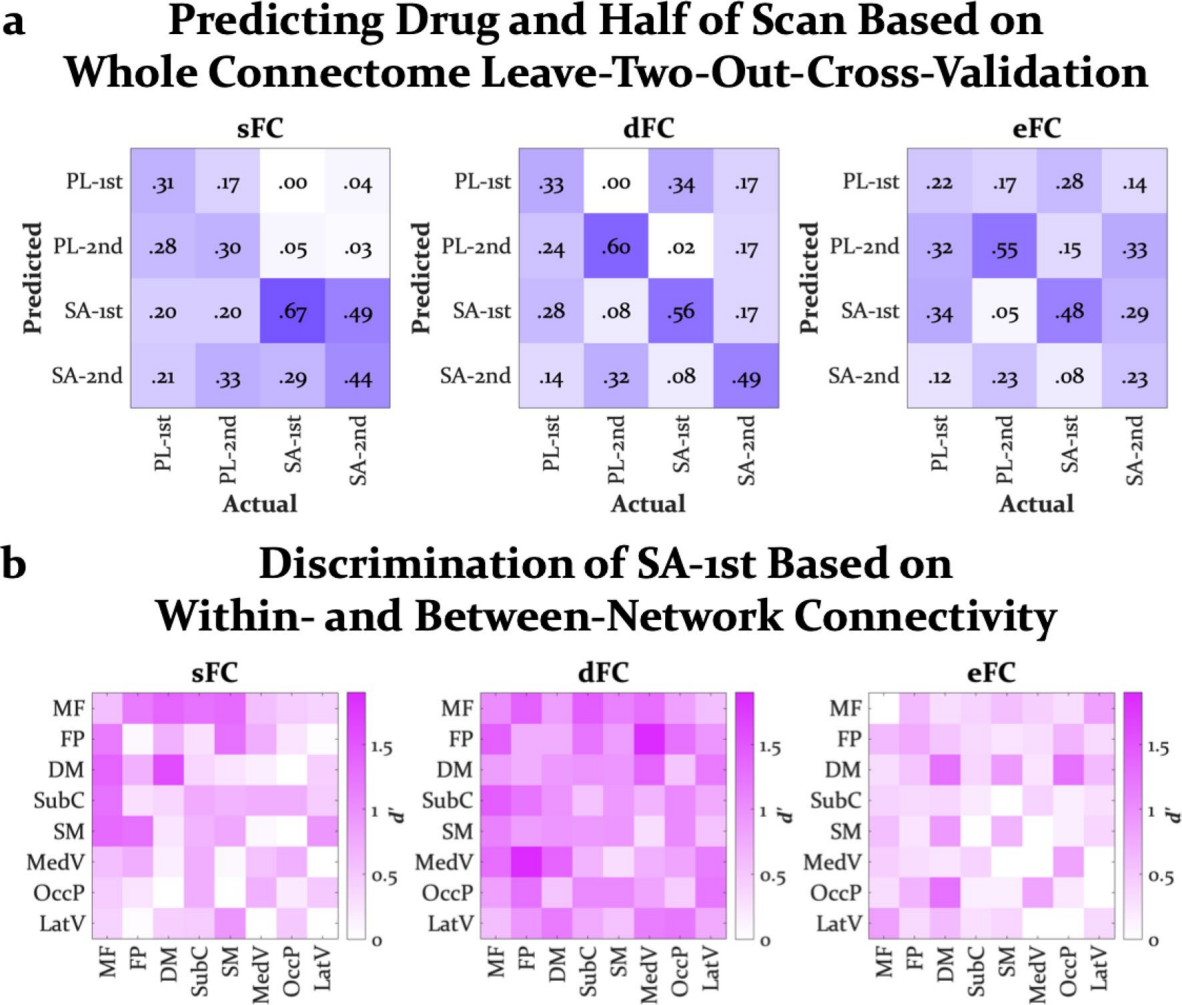
(**a**) Confusion matrices from the leave-two-participant-out cross-validation procedure, training partial least squares models on whole-brain static, dynamic, and entropic connectivity. (**b**) Matrices of discrimination scores (*d*’) of first half salvinorin A scans from the leave-one-participant-out cross-validation procedure, training partial least squares models on within- and between-network static, dynamic, and entropic connectivity. Discrimination below chance was zeroed to limit range of color map. SA = salvinorin A, PL = placebo, sFC = static functional connectivity, dFC = dynamic functional connectivity, eFC = entropic functional connectivity, MF = medial frontal network, FP = frontoparietal network, DM = default mode network, SubC = subcortical-cerebellum network (includes the salience network), SM = somatosensory-motor network, MedV = medial visual network, OccP = occipital pole network, and LatV = lateral visual network.

Classification was mostly successful across conditions (chance = 25%), with confusion occurring in a predictable fashion (Fig. 4a). That is, notable confusions included the first and second half of placebo, when cognitive state would not be expected to change much, and first and second half of SA, when some participants experienced drug effects throughout the scan. Notably, first half of the SA scan, arguably the most distinct state in the scanner, tended to be the most accurately classified, rarely being misclassifed as placebo, and placebo scans were also not frequently misclassified as first half of SA. Nevertheless, classification performance was not equal across connectomes. Models trained on whole-brain sFC, dFC, and eFC correctly identified the first half of SA scans 67%, 56%, and 48% of the time, respectively. Moreover, whereas the model trained on whole-connectome sFC rarely mislabeled SA conditions as placebo, the model trained on whole-connectome dFC was generally more often correct across conditions. Combining across connectomes did not drastically improve classification of SA (see Supplementary Fig. S12).

Classification based only on within- or between-network connectomes was mostly above chance, discriminating the first half of SA from all other conditions (Fig. 4b). However, dFC was by far more accurate with most network interactions performing well. The predictive power of dFC for most within- and between-network models may be partially explained by motion, but this does not seem to be the case for dFC within the DMN (see Supplementary Fig. S7). eFC, which was also influenced by motion, had similar predictive power as sFC. Interestingly, the static and entropic connectomes most predictive of first half of SA, on par with predictive dFC connectomes, involved the DMN, specifically within-network DMN. SA-induced changes in within-network DMN sFC and dFC were not correlated with changes in motion (Supplementary Fig. S6 and S7). Within- and between-network connectomes combined across sFC, dFC, and eFC did not drastically improve discrimination of first half of SA (see Supplementary Fig. S12).

## Discussion

This study used fMRI to measure how inhaled SA alters brain functional connectivity in humans. Similar to classic psychedelics^25,30,33,36^ and dissociative anesthetics^31,32,37^, SA tended to decrease sFC within resting-state networks, especially within the DMN, and increase sFC between these networks. Whereas the brainwide decreases in dFC under SA were less consistent with the increased variance and entropy produced by other hallucinogens^40,42–45^, one study found LSD to decrease fMRI signal variance across the brain, and another study found ketamine to reduce dFC across the brain. Furthermore, SA was found to increase eFC across the brain, though such changes were not found to be statistically significant. Finally, connectome-based classification highlighted the importance of DMN interactions to the effects of SA. Overall, these findings are strikingly similar to those of classic psychedelics and dissociative anesthetics.

This study had several limitations. In order to reduce costs, a multi-session, crossover design was not implemented, which would have minimized possible expectancy and order effects. Furthermore, we did not utilize electrocardiogram and/or a respiration belt measurements that could have been used to control for potential cardiac and respiratory artifacts, though evidence has been shown for the non-inferiority of the nuisance correct method that we implemented (nuisance regression using principal components of signal in white matter and cerebrospinal fluid) over more direct methods of correcting for respiration and cardiac functioning^53^. Finally, to ensure that participants were able to tolerate drug effects in the scanner, we only enlisted experienced hallucinogen users. The final sample of volunteers who both inquired and qualified for this study consisted only of males, thereby limiting generalizability. Additionally, the impact of chronic or extensive hallucinogen use on brain function is not well-understood, and may further limit the generalizability of these findings to potential effects in the brains of those naïve to hallucinogen use.

Another limitation of the present study was the small sample similar to previous first-in-human fMRI studies with classic psychedelics (*N* = 15 in both^25,30^). However, the scans of this study (20 min) were longer than prior work (6 and 14 min), a factor important to the reliability of resting state measures^54^. Additionally, several statistically conservative and complementary approaches were taken to draw conclusions regarding SA effects on brain function. First, this study focused on reliable, large-scale changes in functional connectivity and avoided drawing strong conclusions about cherry-picked functional connections or networks unless the effects were large or predicted a priori. Second, when mapping changes in individual static connections, data were thresholded using a Bonferroni correction for all 35,778 functional connections. Third, for network analyses, every pairwise combination of brain regions was included within a network or between two networks. Although this approach de-emphasizes hub regions, it is a more defensible exploratory approach, as it does not exclude data or arbitrarily choose seed regions. An assumption was that if there was a large signal from a drug manipulation that substantially altered connectivity at a network level, then it should overcome any noise from including all within- or between-network edges. Finally, internal validity of the data was tested using connectome-based classification. Future application of this novel approach using multiple datasets may shed light on which neural mechanisms, such as increased DMN entropy^40^, disruption of thalamocortical circuits^55^, or disruption of claustrocortical circuits^56^ are common among or unique between different hallucinogens. Investigation of the claustrum, a structure thought to contribute to consciousness and delusional states, may especially be a fruitful avenue for future work considering the high expression of 5-HT_2A_, NMDA, and κ-opioid receptors in the claustrum^57–59^ and given recent developments in measuring claustrum function in humans using fMRI^60,61^.

One of the most robust effects of salvinorin A was a decrease in sFC within the DMN, and using connectome-based classification, we found within-DMN sFC and eFC to be especially predictive of the effects of SA on brain function compared to other network interactions. Decreases in DMN sFC during the acute effects of hallucinogens have been a replicable finding, occurring with psilocybin^30,34^, LSD^25,35^, DMT^33^, and ketamine^31,32^. Although it might be tempting to speculate that these effects reflect “ego dissolution”^30,40^, decreases in sFC within the DMN have also been observed with acute administration of drugs not typically associated with ego dissolution such as THC^62^, alcohol^63^, and amphetamine^64^. The small samples, short scan times, numerous reports based on single datasets, underreporting of comparable or larger effects on other brain networks, and inconsistency across studies between brain measures and ego dissolution undermine the strength of reverse inferential claims regarding the specificity of DMN effects to ego dissolution. Furthermore, the wide variety of methods make direct comparisons more challenging. The contribution of hallucinogen imaging data to public repositories will allow for future work to combine across datasets and use homogenous methods to validate and fully interrogate changes produced by hallucinogens in the DMN and other networks^65^.

There was also some evidence that SA decreased within- but increased between-network sFC, especially in the first 10 min of the scan, a particularly prominent pattern of effects observed with LSD^25,35^. These effects were much subtler in our study, perhaps due to the short time course of peak SA effects. Nevertheless, nearly all uncorrected significant effects and 75% of within- and 88% of between-network changes numerically followed this pattern. Furthermore, using an extremely conservative thresholding procedure that largely returned bilateral within-network connections, it was found that several of these static connections were attenuated under SA.

Interestingly, SA decreased brainwide dFC but tended to increase eFC. Both of these changes were correlated to some degree with motion, but there was evidence that motion may have attenuated some of these effects (see Supplementary Fig. S7 and S8). Further evidence that motion could not fully explain these effects comes from the fact that dFC of most between-network interactions was a consistently good predictor of SA effect on brain function, whereas eFC of only selective networks was predictive. Although the decreases in dFC were unexpected in light of the “entropy” produced by hallucinogens, ketamine was similarly found to attenuate but not increase dFC across the brain, especially in visual network interactions^46^. Moreover, despite discussions of variability and entropy in similar terms^40^, these measures should not be expected to be impacted similarly. For example, a functional connection or brain area that fluctuates between a very strong and a very weak state would produce high variability and low entropy. In contrast, an edge that fluctuates between several neighboring connectivity strengths would produce high eFC without necessarily producing high dFC. Therefore, one interpretation of the current data is that SA minimizes drastic changes in connectivity while increasing the number of qualitatively distinct states. Using methods that specifically identify recurring and unique brain states may help support such an interpretation^44,49^.

It is worth noting that the DMN stood out as the best predictor of brain function for sFC and eFC. These findings are somewhat at odds with the specificity of the entropic brain hypothesis, which emphasizes how classic psychedelics drive entropy in the DMN and other higher level networks that supposedly constrain the state-space of the brain^40,41^. By increasing DMN entropy, it has been proposed that psychedelics could loosen the inflexibility of behaviors in conditions such as depression and addiction. If such effects of classic psychedelics are important and related to therapeutic outcomes, then it could be inferred based on our findings that that such benefits extend to κ-opioid agonists. Indeed, SA and other κ-opioid agonists may be promising treatments for depression^14,15^ and cocaine addiction^20^ (but see^66^), and ibogaine, a compound with a much longer time course than SA and both κ-opioid and 5-HT_2A_ agonist activity, is being explored for the treatment of addiction and mood disorders^18,19^. With the increasing acceptance of hallucinogens as potential therapeutics, exploration of different pharmacological tools and their combinations will be a necessary avenue in future research.

### Data availability

Data can be made available to qualified research institutions upon reasonable request and data use agreement executed with Johns Hopkins University.

## Supplementary information

Supplementary file1
