# Supplementary material for: The Acute Effects of the Atypical Dissociative Hallucinogen Salvinorin A on Functional Connectivity in the Human Brain
Source: Sci Rep. 2020 Oct 2;10:16392. doi: 10.1038/s41598-020-73216-8 (PMC7532139; doi:10.1038/s41598-020-73216-8)
Supplement: Supplementary file 1 — Supplementary file1 [file 41598_2020_73216_MOESM1_ESM.docx]

**Supplementary Information for**

**The Acute Effects of the Atypical Dissociative Hallucinogen Salvinorin A on**

**Functional Connectivity in the Human Brain**

Manoj K. Doss^1^*, Darrick G. May^1^, Matthew W. Johnson^1^, John M. Clifton^1^, Sidnee L. Hedrick^2^, Thomas E. Prisinzano^2^, Roland R. Griffiths^1,3^, & Frederick S. Barrett^1^

^1^Department of Psychiatry and Behavioral Sciences, Center for Psychedelic & Consciousness Research, Johns Hopkins University School of Medicine

^2^Department of Pharmaceutical Sciences, College of Pharmacy, University of Kentucky

^3^Department of Neuroscience, Johns Hopkins University School of Medicine

*Correspondence to: Manoj K. Doss, 5510 Nathan Shock Drive, Baltimore, MD 21224

Phone: 410-550-5953

Email: mdoss3@jhmi.edu

Key words: salvinorin A, *Salvia divinorum*, hallucinogen, psychedelic, fMRI, functional connectivity

**Supplementary Information**

**Methods**

*Drug and Delivery Device*

The dose, device, and administration procedure were all based on the methods used in our previous study and shown to induce moderately high subjective effects ^1,2^. Salvinorin A was isolated from *Salvia divinorum* by one of the authors (T.E.P.), and a pharmacist prepared individual doses of 15 µg/kg of salvinorin A in an acetone solution that was dropped into the bottom of a 5 ml round-bottom flask with acetone subsequently evaporated at room temperature. This produced a small visible quantity of salvinorin A in the bottom of the flask. The drug delivery device consisted of this flask (containing 15 µg/kg salvinorin A or nothing for placebo) that was secured to a chemistry vacuum adapter using a metal Keck clip. Polytetrafluoroethylene tubing (.635 cm inner diameter) was connected to the adapter’s vacuum line for inhalation. The tube length was 400 cm for half the participants and 690 cm for the other half, as there were differences in the distance from the control room to scanner at imaging sites. Tubing was replaced between participants. In simulated inhalations of salvinorin A, approximately 41.06% of drug remained in the 690 cm tube (see below analysis of salvinorin A deposition in tubes).

*Fahn-Tolosa-Marin Tremor Rating Scale*

The Tremor Rating Scale (Fahn et al., 1993), used previously to classify resting and kinetic tremor severity in our dose-effects study of salvinorin A (Johnson et al., 2011), was assessed immediately before, as well as 15 and 30 minutes after salvinorin A administration. At these time points, session monitors visually assessed kinetic tremor intensity as the participant bent both of their extended arms to touch middle finger to nose. Study staff also rated the magnitude of resting tremor during the time interval leading up to the assessments of kinetic tremor (i.e., 0-15 min,15-30 min) by closely observing the volunteer. There were no tremors, and therefore, no analyses of tremors are reported.

*Imaging Parameters and Preprocessing*

The first six volunteers were scanned on a 3T Siemens Skyra at the Center for Translational Molecular Imaging, but due to a scanner failure (not while scanning), the second six volunteers were scanned on a 3T Siemens Prisma at the MRI Service Center (both at Johns Hopkins University). Both scanners used a 32-channel headcoil, and pulse sequences were identical between scanners. At the scanning session for each subject, the first scan was a T1-weighted structural MPRAGE (TR = 2300 ms, TE = 3.01 ms, flip angle = 9°, acquisition matrix = 240 × 240 mm, in-plane resolution = .9 × .9 mm, slice thickness = 1 mm). The following scan was a diffusion-weighted imaging scan, but these data are not reported here. The next two scans used multiband echo-planar imaging to measure blood-oxygenation level-dependent (BOLD) fMRI (1320 TRs, TR = 907 ms, total acquisition time = 20 minutes, TE = 43 ms, flip angle = 52°, acquisition matrix = 224 × 224 mm, voxel size = 2 mm^3^, 66 axial-oblique slices parallel to the anterior/posterior commissure line, multiband acceleration factor = 6).

Spatial preprocessing of functional images was performed in SPM12 and included realignment (motion correction), coregistration of the second BOLD scan with the first BOLD scan, normalization of BOLD scans to a standard MNI template ^3^ using a 4^­^th degree B-Spline interpolation, and smoothing with a 6 mm full width at half maximum Gaussian kernel. Temporal preprocessing was performed using tools from the Cognitive and Affective Neuroscience Lab (http://github.com/canlab) and included simultaneous bandpass filtering (.009-.08 Hz) and nuisance regression. Nuisance parameters consisted of linear trend, the first 5 principle components of voxels containing cerebrospinal fluid and the first 5 principle components of voxels containing white matter signal (both identified using masks derived from segmented and normalized T1-weighted structural images ^4^), 24 motion parameters from realignment (translations and rotations, their derivatives, and squares of all of these ^5^) and motion censoring or “scrubbing” regressors ^6^ generated from the ART toolbox using outlier detection and intermediate settings (global-signal *z*-value threshold = 5, subject-motion threshold = 0.9 mm). Finally, these data were parcellated using the Shen atlas ^7^ by averaging voxels within each of the 268 atlas regions at each time point to produce 268 timeseries. These atlas regions can be organized into the following eight networks: medial frontal (MF), frontoparietal (FP), default mode (DM), subcortical-cerebellum (SubC; includes the salience network), somatosensory-motor (SM), medial visual (MedV), occipital pole (OccP), and lateral visual (LatV).

*Questionnaires Completed at the End of Each Session*

See Table S1 for effects of salvinorin A from each session on the following questionnaires.

Mystical Experience Questionnaire (MEQ30)

The MEQ30 is a 30-item questionnaire developed to assess phenomenological content during altered states of consciousness ^8,9^. It has been shown to be sensitive to the effects of salvinorin A and classic psychedelics. Items are rated on a 6-point scale ranging from 0 (none, not at all) to 5 (extreme, more than ever before in my life). In addition to a total score, the MEQ30 is composed of four factors: mystical, positive mood, transcendence of time and space, and ineffability. Scores are expressed as percentages of items endorsed.

Challenging Experience Questionnaire (CEQ)

The CEQ is 26-item questionnaire developed to assess challenging aspects of hallucinogen experiences ^10^. Items are rated on a 6-point scale ranging from 0 (none, not at all) to 5 (extreme, more than ever before in my life and stronger than 4). In addition to a total score, the CEQ is composed of seven factors: fear, grief, physical distress, insanity, isolation, death, paranoia. Scores are expressed as percentages of items endorsed.

Multi-Dimensional Assessment for Interoceptive Awareness (MAIA)

The MAIA is a 32-item questionnaire designed to measure interoceptive awareness (i.e., awareness of bodily processes) ^11^. It has been shown to be sensitive to the effects of salvinorin A ^12^. Items are rated on a 6-point scale ranging from 0 (never) to 5 (always). The MAIA is composed of eight factors: noticing, not distracting, not worrying, attention regulation, emotional awareness, self-regulation, body listening, trusting.

Five-Dimensional Altered States of Consciousness Questionnaire (5D-ASC)

The 5D-ASC is a 94-item questionnaire designed to measure altered states of consciousness ^13^. It has been shown to be sensitive to the effects of psilocybin and ketamine. Items are rated on a 20-point scale ranging from 1 (no, not more than usually) to 20 (yes, much more than usually). The 5D-ASC is composed of 11 factors: experience of unity, spiritual experience, blissful state, insightfulness, disembodiment, impaired control and cognition, anxiety, complex imagery, elementary imagery, audio-visual synesthesia, changed meaning of percepts.

Visual Analog Scale (VAS)

The VAS was used to retrospectively rate peak effects and abuse liability of salvinorin A. Each of the 12 VAS items were rated on a sliding scale from 0 (no effects) to 100 (the most extreme imaginable). These items were overall drug effects, good effects, bad effects, sudden start of effects, fear, change in perception, loss of body, loss of contact with external reality, visual effects, auditory effects, liking, and take again.

Realism Item

This questionnaire contains three statements that retrospectively gauged how “real” the experience of salvinorin A seemed. Statements were presented on a sliding scale from 0 to 100.

Psychological Insight Questionnaire

This 29-item questionnaire was designed to probe supposed insights had during or after salvinorin A. Items are rated on a 6-point scale ranging from 0 (none) to 5 (extreme). Items are averaged into a single score and are expressed as percentages.

**Results**

*Analysis of Salvinorin A Deposition in Tubes*

To estimate the amount of drug actually delivered through the PFTE tubing, the inhalation procedure was simulated with 23 cm and 690 cm tubes using a vacuum syringe and the tubes were analyzed for deposition content. In this simulation, a glass bulb containing the highest dose of salvinorin A used in the study (1.6 mg) was attached to the PTFE tube, and the tube was attached to a 3 L vacuum syringe. While heating the glass bulb with butane torches, the syringe was slowly pulled over 45 seconds, simulating an inhalation. Upon completion, there was no salvinorin A remaining in the glass bulb. The simulation was performed a total of 6 times (3 times with a 23 cm tube, 3 times with a 690 cm tube). The tubes were immediately sealed with parafilm and tape, then sent to the University of Kentucky for deposition analysis.

To determine the percent dose of salvinorin A remaining in the PTFE tubes, the tubes were washed with methanol (10 mL per 23 cm tube, 300 mL per 690 cm tube), the wash was concentrated *in vacuo*, and the mass of residual salvinorin A was calculated. For the 23 cm tubes, an average of 0.077 ± 0.029 mg of salvinorin A remained in the tube (4.81% of the dose). For the 690 cm tubes, an average of 0.657 ± 0.198 mg of salvinorin A remained in the tube (41.06%).

To confirm the chemical identity of salvinorin A after washing, the resulting residue was characterized using mass spectrometry (Advion expression CMS-S). The residue had identical ion spectra to an authenticated standard of salvinorin A. In all cases, salvinorin A was present in >99% purity (Fig. S1).


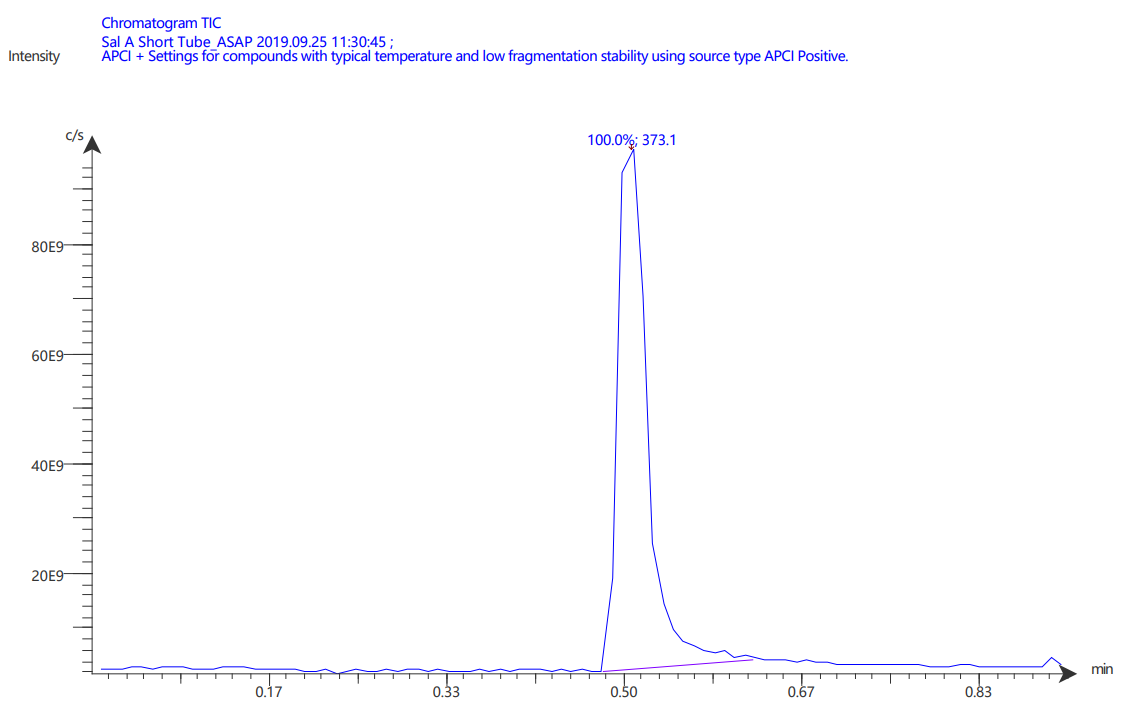

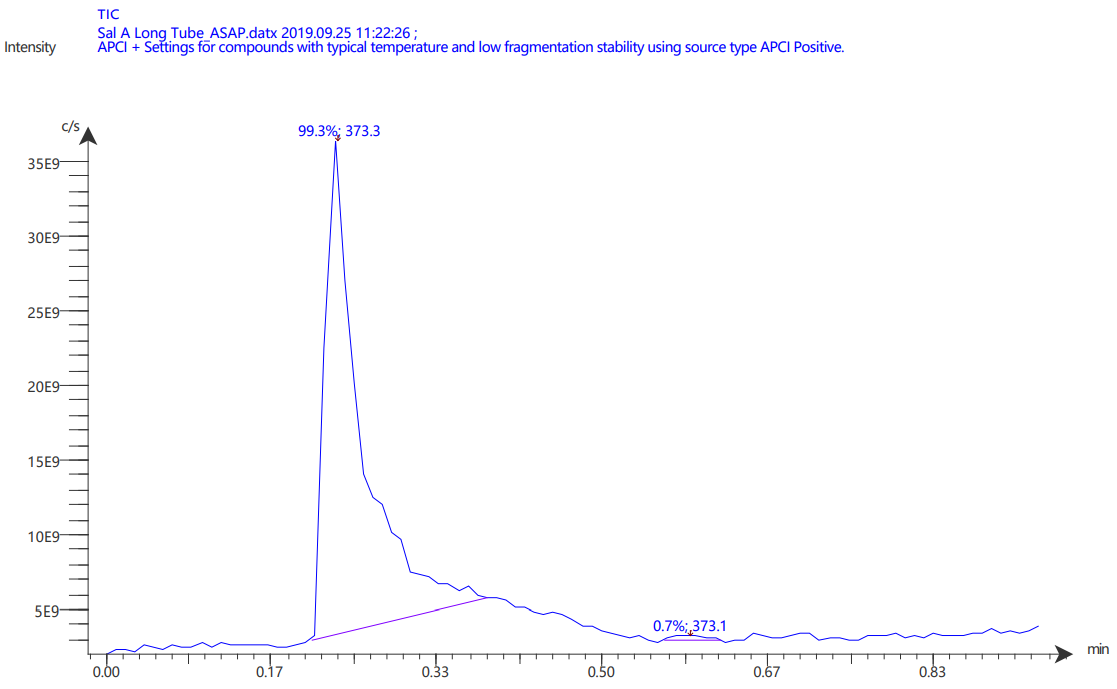


**Figure S1.** Representative CMS traces for the 23 cm tube (top) and 690 cm tube (bottom).

*Reliability of First and Second Half of Scans*

As a sanity check, the reliability of static, dynamic, and entropic functional connectivity (sFC, dFC, and eFC, respectively) was tested by correlating edges during the first half of a scan with edges during the second half a scan in each subject. Fig. S2a-c plots every edge of every participant for each measure in each drug condition. In both drug conditions, reliability was high for static (placebo: *r* = .76; salvinorin A: *r* = .69), dynamic (placebo: *r* = .81; salvinorin A: *r* = .86), and entropic (placebo: *r* = .62; salvinorin A: *r* = .76) functional connectivity. Compared to static and dynamic connectivity, entropic connectivity data were less clustered and contained a handful of outliers (values far below 0). However, even these values seemed to be consistent between the first and second half of scans.

Although aggregating the data across participants in this fashion did not reveal large differences between drug conditions, plotting individual participant correlations did (Fig. S2d). In the case of static functional connectivity, despite high *r*-values in all participants in both drug conditions, it can be seen that the reliability between the first and second half of scans of every participant is numerically reduced in the salvinorin A scan. This decrease in reliability may come from the fact that participants experienced peak drug effects in the first 10 minutes but much weaker effects (or none at all) in the second half. In contrast to static connectivity, entropic functional connectivity was less reliable in some participants, and interestingly, reliability seemed to be greater in the salvinorin A scan. This could be explained by the fact that entropic functional connectivity tended to decrease within a scan, especially in the placebo scan (see Table S2 for ANOVA statistics), perhaps reflecting participant fatigue ^14,15^. In contrast, lingering salvinorin A-induced increases in entropic functional connectivity during the second half of the salvinorin A scan could produce more stable measures of entropic functional connectivity compared to the placebo scan. There were no noticeable between-drug differences in the reliability of dynamic functional connectivity, which was overall the most reliable measure of functional connectivity.

**
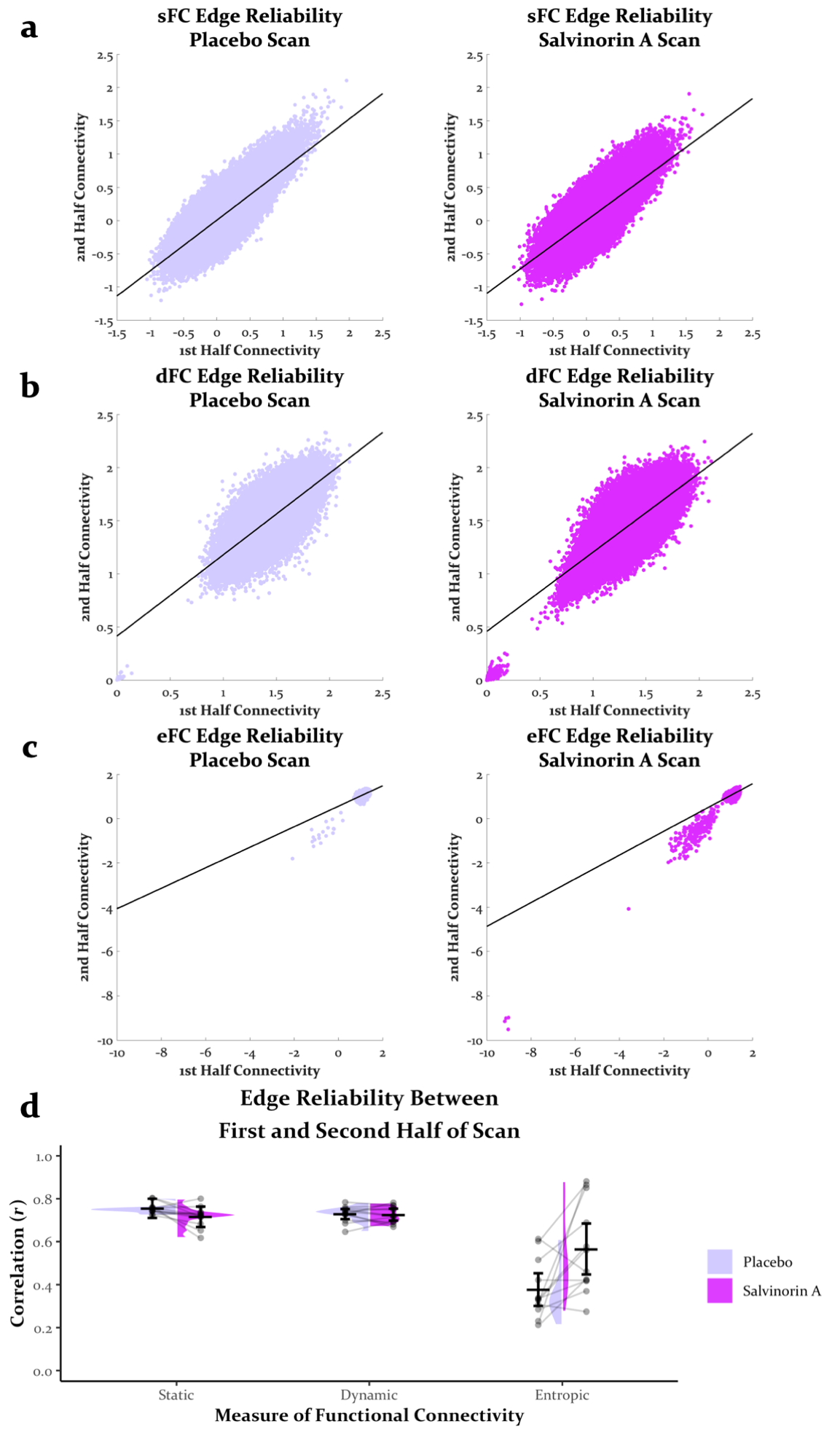
**

**Figure S2.** Reliability between the first and second half of scans for (**a**) static, (**b**) dynamic, and (**c**) entropic functional connectivity. Each point represents a single edge for an individual subject. (**d**) Violin plots of individual participant reliability (*r*-values) of functional connectivity measures.


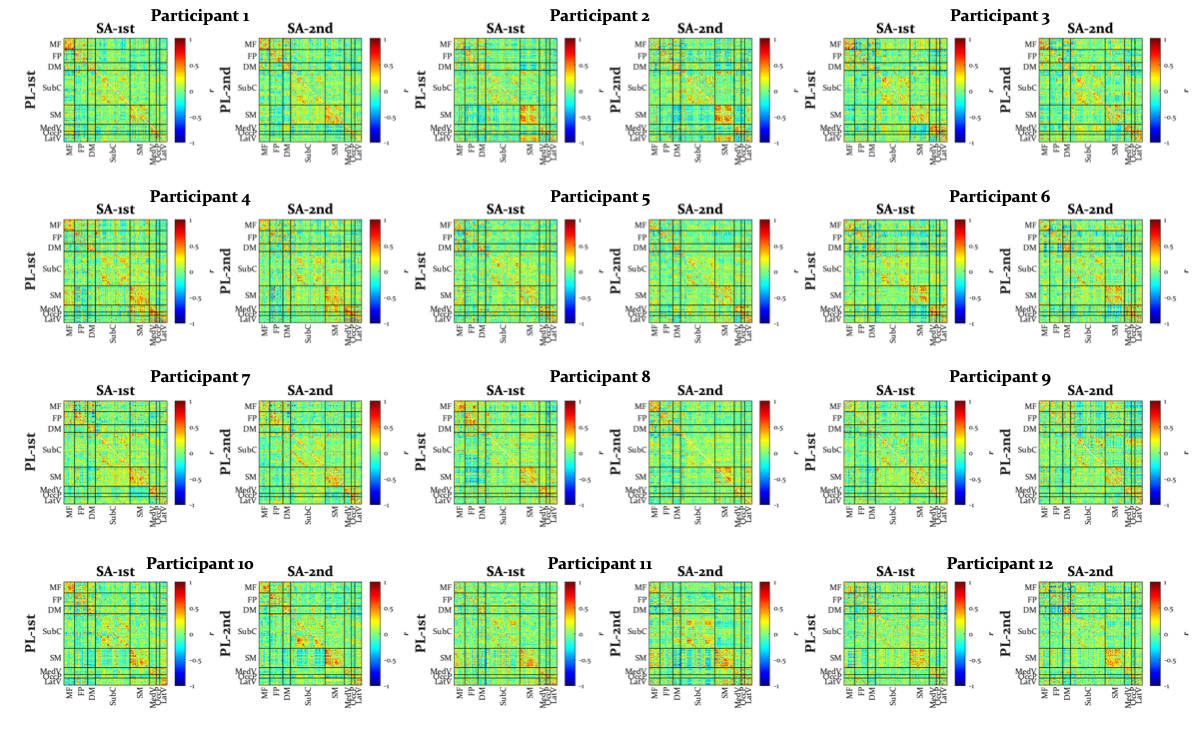


**Figure S3.** Single participant static functional connectivity matrices. SA = salvinorin A, PL = placebo, MF = medial frontal network, FP = frontoparietal network, DM = default mode network, SubC = subcortical-cerebellum network (includes the salience network), SM = somatosensory-motor network, MedV = medial visual network, OccP = occipital pole network, and LatV = lateral visual network.


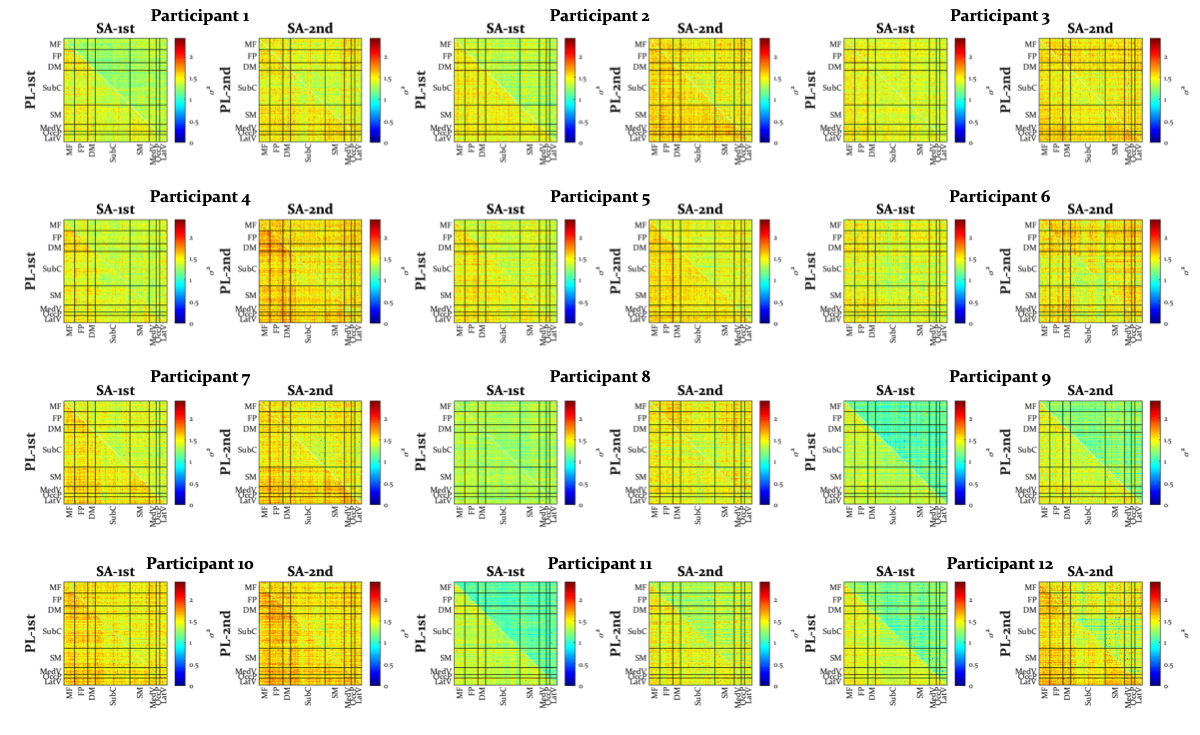


**Figure S4.** Single participant dynamic functional connectivity matrices. SA = salvinorin A, PL = placebo, MF = medial frontal network, FP = frontoparietal network, DM = default mode network, SubC = subcortical-cerebellum network (includes the salience network), SM = somatosensory-motor network, MedV = medial visual network, OccP = occipital pole network, and LatV = lateral visual network.


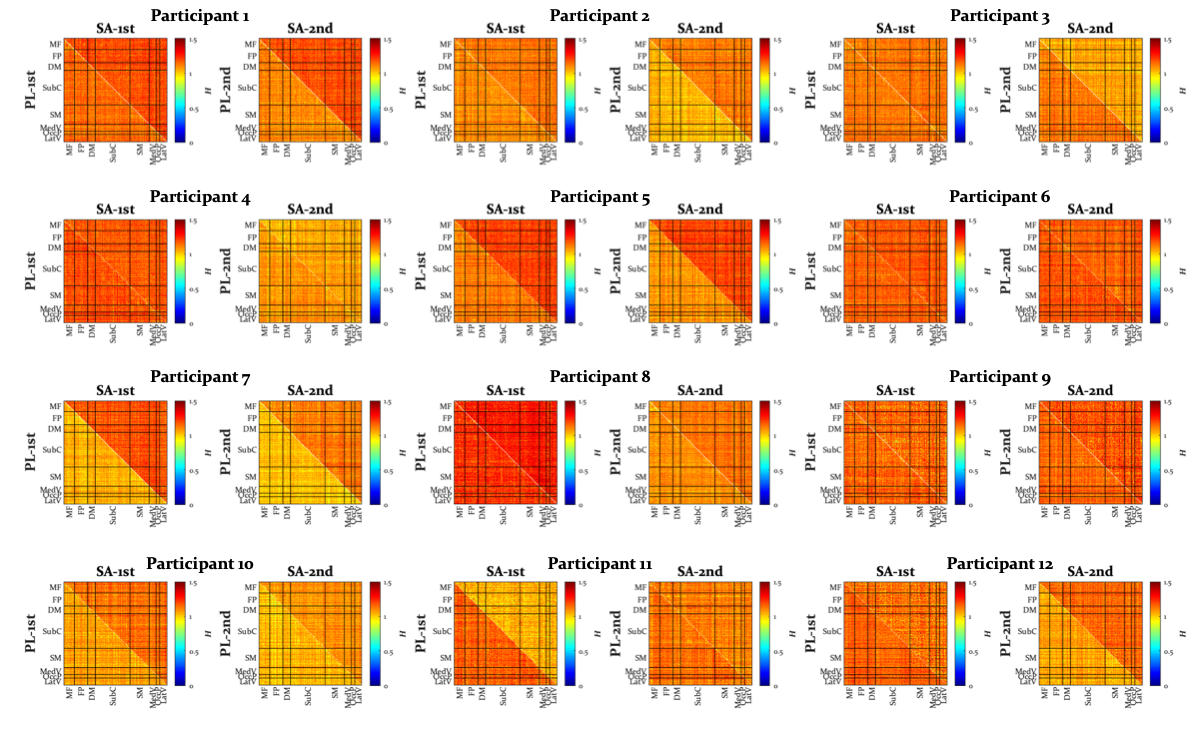


**Figure S5.** Single participant entropic functional connectivity matrices. SA = salvinorin A, PL = placebo, MF = medial frontal network, FP = frontoparietal network, DM = default mode network, SubC = subcortical-cerebellum network (includes the salience network), SM = somatosensory-motor network, MedV = medial visual network, OccP = occipital pole network, and LatV = lateral visual network.

*Questionnaire Data*

**Table S1.** Mean and 95% confidence interval of subjective ratings completed at the end of both administrations of salvinorin A. MEQ = Mystical Experience Questionnaire, CEQ = Challenging Experience Questionnaire, MAIA = Multi-Dimensional Assessment for Interoceptive Awareness, 5D-ASC = Five-Dimensional Altered States of Consciousness, VAS = Visual Analog Scale.

|  | Practice Session | Scanning Session |
| --- | --- | --- |
| MEQ30 Total | .51 [.40, .61] | .49 [.35, .62] |
| MEQ30 Mystical | .44 [.30, .58] | .40 [.22, .58] |
| MEQ30 Positive Mood | .49 [.35, .63] | .44 [.30, .59] |
| MEQ30 Transcendence | .64 [.53, .76] | .68 [.56, .80] |
| MEQ30 Ineffability | .59 [.41, .78] | .63 [.46, .80] |
| CEQ Fear | .90 [.29, 1.51] | .98 [.36, 1.60] |
| CEQ Grief | .17 [-.04, .37] | .38 [-.01, .76] |
| CEQ Physical Distress | .68 [.33, 1.04] | .67 [.23, 1.10] |
| CEQ Insanity | .89 [.12, 1.66] | 1.14 [.38, 1.90] |
| CEQ Isolation | 1.22 [.40, 2.04] | 1.19 [.37, 2.02] |
| CEQ Death | .33 [-.18, .85] | .79 [.06, 1.53] |
| CEQ Paranoia | .21 [-.11, .52] | .33 [-.06, .72] |
| CEQ Total | .63 [.26, 1.00] | .76 [.42, 1.10] |
| MAIA Noticing | 1.56 [.65, 2.48] | 2.00 [1.02, 2.98] |
| MAIA Not Distract | 4.67 [4.33, 5.00] | 3.89 [3.09, 4.68] |
| MAIA Not Worrying | 3.56 [3.25, 3.86] | 3.72 [3.28, 4.16] |
| MAIA Attention Regulation | 2.08 [1.20, 2.97] | 1.55 [.74, 2.35] |
| MAIA Emotional Awareness | 1.80 [1.07, 2.53] | 1.22 [.44, 1.99] |
| MAIA Self-Regulation | 1.94 [1.12, 2.75] | 1.48 [.68, 2.28] |
| MAIA Body Listening | 1.50 [.84, 2.16] | .81 [.07, 1.54] |
| MAIA Trusting | 2.78 [1.72, 3.84] | 2.47 [1.53, 3.41] |
| 5D-ASC Experience of Unity | 49.52 [33.99, 65.05] | 39.60 [19.67, 59.53] |
| 5D-ASC Spiritual Experience | 37.72 [20.15, 55.29] | 42.89 [22.72, 63.06] |
| 5D-ASC Blissful State | 32.86 [12.00, 53.72] | 33.25 [13.89, 52.61] |
| 5D-ASC Insightfulness | 18.72 [9.37, 28.08] | 23.39 [13.00, 33.77] |
| 5D-ASC Disembodiment | 52.86 [33.81, 71.91] | 58.69 [38.25, 79.14] |
| 5D-ASC Impaired Control | 15.75 [2.77, 28.73] | 21.35 [8.05, 34.64] |
| 5D-ASC Anxiety | 13.58 [4.45, 22.72] | 24.99 [13.20, 36.77] |
| 5D-ASC Complex Imagery | 37.19 [18.44, 55.95] | 40.14 [20.04, 60.24] |
| 5D-ASC Elementary Imagery | 49.53 [25.85, 73.21] | 53.44 [30.79, 76.10] |
| 5D-ASC Synesthesia | 10.19 [-3.57, 23.96] | 17.47 [-1.65, 36.59] |
| 5D-ASC Changed Percepts | 10.03 [1.81, 18.25] | 8.03 [-1.43, 17.49] |
| VAS Overall Drug Effects | 79.50 [68.70, 90.30] | 89.08 [81.24, 96.93] |
| VAS Good Effects | 74.42 [59.88, 88.95] | 68.42 [50.98, 85.86] |
| VAS Bad Effects | 10.92 [2.46, 19.37] | 15.17 [7.24, 23.10] |
| VAS Sudden Start of Effects | 72.92 [54.44, 91.39] | 78.17 [64.53, 91.80] |
| VAS Fear | 10.58 [2.04, 19.12] | 16.50 [5.64, 27.36] |
| VAS Change in Perception | 68.25 [52.06, 84.44] | 60.17 [38.42, 81.91] |
| VAS Loss of Body | 47.67 [25.37, 69.96] | 69.08 [51.13, 87.04] |
| VAS Loss of External Reality | 66.67 [44.02, 89.31] | 80.58 [62.85, 98.32] |
| VAS Visual Effects | 69.92 [49.65, 90.18] | 63.92 [41.93, 85.90] |
| VAS Auditory Effects | 12.58 [-.42, 25.59] | 18.33 [-1.38, 38.05] |
| VAS Liking | 71.67 [54.78, 88.55] | 64.08 [45.84, 82.33] |
| VAS Take Again | 75.75 [58.99, 92.51] | 72.92 [56.03, 89.80] |
| Realism 1 | 59.67 [35.56, 83.77] | 50.50 [22.80, 78.20] |
| Realism 2 | 18.08 [-.82, 36.98] | 21.75 [-.43, 43.93] |
| Realism 3 | 34.83 [11.04, 58.63] | 47.58 [23.32, 71.84] |
| Insight Score | .61 [.14, 1.08] | .57 [.22, .91] |

*ANOVA Results for Within- and Between-Network Effects of Salvinorin A*

**Table S2.** Drug (placebo, salvinorin A) by time (first half, second half) ANOVAs on within- and between-network static, dynamic, and entropic functional connectivity. sFC = static functional connectivity, dFC = dynamic functional connectivity, eFC = entropic functional connectivity, MF = medial frontal network, FP = frontoparietal network, DM = default mode network, SubC = subcortical-cerebellum network (includes the salience network), SM = somatosensory-motor network, MedV = medial visual network, OccP = occipital pole network, and LatV = lateral visual network.

|  |  | sFC | | | dFC | | | eFC | | |
| --- | --- | --- | --- | --- | --- | --- | --- | --- | --- | --- |
| Network(s) | Effect | *F*(1,11) | *p* | $\eta_{p}^{2}$ | *F*(1,11) | *p* | $\eta_{p}^{2}$ | *F*(1,11) | *p* | $\eta_{p}^{2}$ |
| MF | Drug | .40 | .540 | .04 | 12.11 | .005 | .52 | 4.04 | .069 | .27 |
|  | Time | .12 | .732 | .01 | 33.48 | .000 | .75 | 7.45 | .020 | .40 |
|  | Drug × Time | .54 | .477 | .05 | 46.97 | .000 | .81 | .94 | .354 | .08 |
| FP | Drug | 5.89 | .034 | .35 | 14.09 | .003 | .56 | 3.13 | .104 | .22 |
|  | Time | .27 | .611 | .02 | 29.65 | .000 | .73 | 5.41 | .040 | .33 |
|  | Drug × Time | 1.30 | .279 | .11 | 28.47 | .000 | .72 | .42 | .531 | .04 |
| DM | Drug | 9.40 | .011 | .46 | 12.98 | .004 | .54 | 3.46 | .090 | .24 |
|  | Time | 7.30 | .021 | .40 | 44.43 | .000 | .80 | 11.67 | .006 | .51 |
|  | Drug × Time | 3.50 | .088 | .24 | 12.43 | .005 | .53 | .60 | .454 | .05 |
| SubC | Drug | 7.77 | .018 | .41 | 12.20 | .005 | .53 | 3.48 | .089 | .24 |
|  | Time | .14 | .715 | .01 | 40.29 | .000 | .79 | 6.42 | .028 | .37 |
|  | Drug × Time | 3.06 | .108 | .22 | 3.55 | .086 | .24 | 1.92 | .193 | .15 |
| SM | Drug | 2.62 | .134 | .19 | 9.73 | .010 | .47 | 4.05 | .069 | .27 |
|  | Time | 1.02 | .335 | .08 | 58.01 | .000 | .84 | 9.42 | .011 | .46 |
|  | Drug × Time | 1.60 | .231 | .13 | 3.03 | .110 | .22 | .43 | .527 | .04 |
| MedV | Drug | 5.86 | .034 | .35 | 12.23 | .005 | .53 | 1.75 | .213 | .14 |
|  | Time | .59 | .458 | .05 | 64.99 | .000 | .86 | 12.37 | .005 | .53 |
|  | Drug × Time | .01 | .941 | .00 | .96 | .348 | .08 | 1.88 | .198 | .15 |
| OccP | Drug | .38 | .551 | .03 | 1.99 | .186 | .15 | 1.91 | .195 | .15 |
|  | Time | .40 | .540 | .04 | 39.71 | .000 | .78 | 3.62 | .084 | .25 |
|  | Drug × Time | 3.29 | .097 | .23 | .14 | .715 | .01 | .34 | .571 | .03 |
| LatV | Drug | .27 | .613 | .02 | 11.80 | .006 | .52 | 3.01 | .111 | .21 |
|  | Time | .31 | .588 | .03 | 70.18 | .000 | .86 | 8.42 | .014 | .43 |
|  | Drug × Time | .88 | .370 | .07 | 4.00 | .071 | .27 | 7.38 | .020 | .40 |
| MF-FP | Drug | .31 | .590 | .03 | 17.33 | .002 | .61 | 3.40 | .092 | .24 |
|  | Time | .16 | .698 | .01 | 32.93 | .000 | .75 | 6.34 | .029 | .37 |
|  | Drug × Time | 3.30 | .097 | .23 | 34.55 | .000 | .76 | .57 | .467 | .05 |
| MF-DM | Drug | 2.48 | .144 | .18 | 19.96 | .001 | .64 | 2.94 | .114 | .21 |
|  | Time | .39 | .545 | .03 | 47.07 | .000 | .81 | 10.14 | .009 | .48 |
|  | Drug × Time | 1.58 | .234 | .13 | 19.02 | .001 | .63 | .61 | .451 | .05 |
| MF-SubC | Drug | .00 | .977 | .00 | 15.19 | .002 | .58 | 3.21 | .101 | .23 |
|  | Time | .05 | .835 | .00 | 37.96 | .000 | .78 | 6.93 | .023 | .39 |
|  | Drug × Time | .00 | .984 | .00 | 14.55 | .003 | .57 | 1.47 | .250 | .12 |
| MF-SM | Drug | .21 | .656 | .02 | 12.77 | .004 | .54 | 3.76 | .078 | .25 |
|  | Time | .00 | .958 | .00 | 48.23 | .000 | .81 | 7.87 | .017 | .42 |
|  | Drug × Time | .28 | .608 | .02 | 18.70 | .001 | .63 | .79 | .394 | .07 |
| MF-MedV | Drug | .36 | .562 | .03 | 15.82 | .002 | .59 | 2.48 | .144 | .18 |
|  | Time | 2.74 | .126 | .20 | 64.87 | .000 | .86 | 9.07 | .012 | .45 |
|  | Drug × Time | .16 | .693 | .01 | 7.00 | .023 | .39 | 1.44 | .255 | .12 |
| MF-OccP | Drug | 5.06 | .046 | .32 | 8.54 | .014 | .44 | 2.97 | .113 | .21 |
|  | Time | .00 | .975 | .00 | 46.04 | .000 | .81 | 7.43 | .020 | .40 |
|  | Drug × Time | .00 | .976 | .00 | 21.46 | .001 | .66 | 2.61 | .134 | .19 |
| MF-LatV | Drug | 4.80 | .051 | .30 | 14.03 | .003 | .56 | 3.04 | .109 | .22 |
|  | Time | .01 | .917 | .00 | 56.85 | .000 | .84 | 8.00 | .016 | .42 |
|  | Drug × Time | 3.30 | .097 | .23 | 11.36 | .006 | .51 | 2.46 | .145 | .18 |
| FP-DM | Drug | 4.23 | .064 | .28 | 20.41 | .001 | .65 | 2.34 | .154 | .18 |
|  | Time | .55 | .472 | .05 | 44.09 | .000 | .80 | 8.08 | .016 | .42 |
|  | Drug × Time | 2.04 | .181 | .16 | 17.30 | .002 | .61 | .60 | .456 | .05 |
| FP-SubC | Drug | .13 | .727 | .01 | 17.34 | .002 | .61 | 3.07 | .108 | .22 |
|  | Time | .67 | .431 | .06 | 35.82 | .000 | .77 | 5.76 | .035 | .34 |
|  | Drug × Time | 1.11 | .314 | .09 | 18.15 | .001 | .62 | 1.32 | .274 | .11 |
| FP-SM | Drug | 2.00 | .184 | .15 | 16.15 | .002 | .59 | 3.30 | .096 | .23 |
|  | Time | .96 | .349 | .08 | 40.66 | .000 | .79 | 6.80 | .024 | .38 |
|  | Drug × Time | 1.01 | .338 | .08 | 22.13 | .001 | .67 | .65 | .437 | .06 |
| FP-MedV | Drug | 1.18 | .300 | .10 | 20.22 | .001 | .65 | 2.52 | .141 | .19 |
|  | Time | 1.87 | .198 | .15 | 61.42 | .000 | .85 | 8.03 | .016 | .42 |
|  | Drug × Time | .02 | .878 | .00 | 10.72 | .007 | .49 | 1.18 | .301 | .10 |
| FP-OccP | Drug | 4.07 | .069 | .27 | 8.17 | .016 | .43 | 2.58 | .136 | .19 |
|  | Time | .02 | .892 | .00 | 56.37 | .000 | .84 | 5.88 | .034 | .35 |
|  | Drug × Time | 2.44 | .147 | .18 | 45.81 | .000 | .81 | 2.39 | .150 | .18 |
| FP-LatV | Drug | 1.37 | .266 | .11 | 16.44 | .002 | .60 | 3.83 | .076 | .26 |
|  | Time | .03 | .867 | .00 | 53.81 | .000 | .83 | 6.41 | .028 | .37 |
|  | Drug × Time | .02 | .893 | .00 | 14.26 | .003 | .56 | 2.25 | .162 | .17 |
| DM-SubC | Drug | 2.94 | .115 | .21 | 14.55 | .003 | .57 | 3.92 | .073 | .26 |
|  | Time | .01 | .924 | .00 | 53.82 | .000 | .83 | 8.36 | .015 | .43 |
|  | Drug × Time | 1.36 | .267 | .11 | 11.29 | .006 | .51 | 1.25 | .287 | .10 |
| DM-SM | Drug | 1.94 | .192 | .15 | 15.70 | .002 | .59 | 3.37 | .094 | .23 |
|  | Time | .53 | .484 | .05 | 54.97 | .000 | .83 | 9.62 | .010 | .47 |
|  | Drug × Time | .34 | .570 | .03 | 21.59 | .001 | .66 | .88 | .367 | .07 |
| DM-MedV | Drug | .03 | .869 | .00 | 24.04 | .000 | .69 | 1.46 | .252 | .12 |
|  | Time | .56 | .471 | .05 | 68.07 | .000 | .86 | 11.51 | .006 | .51 |
|  | Drug × Time | 5.22 | .043 | .32 | 8.04 | .016 | .42 | 1.82 | .205 | .14 |
| DM-OccP | Drug | .10 | .759 | .01 | 8.10 | .016 | .42 | 3.38 | .093 | .24 |
|  | Time | .25 | .625 | .02 | 71.54 | .000 | .87 | 8.69 | .013 | .44 |
|  | Drug × Time | 4.13 | .067 | .27 | 12.17 | .005 | .53 | 1.79 | .208 | .14 |
| DM-LatV | Drug | .52 | .488 | .04 | 15.71 | .002 | .59 | 2.72 | .127 | .20 |
|  | Time | 6.77 | .025 | .38 | 58.27 | .000 | .84 | 10.49 | .008 | .49 |
|  | Drug × Time | 1.44 | .256 | .12 | 9.92 | .009 | .47 | 2.71 | .128 | .20 |
| SubC-SM | Drug | .01 | .921 | .00 | 13.71 | .003 | .55 | 2.99 | .112 | .21 |
|  | Time | 3.33 | .095 | .23 | 49.46 | .000 | .82 | 7.22 | .021 | .40 |
|  | Drug × Time | .56 | .469 | .05 | 5.49 | .039 | .33 | 1.38 | .265 | .11 |
| SubC-MedV | Drug | .74 | .408 | .06 | 14.91 | .003 | .58 | 2.78 | .124 | .20 |
|  | Time | .61 | .452 | .05 | 67.46 | .000 | .86 | 8.91 | .012 | .45 |
|  | Drug × Time | .31 | .591 | .03 | 1.70 | .219 | .13 | 2.09 | .176 | .16 |
| SubC-OccP | Drug | .65 | .438 | .06 | 6.56 | .026 | .37 | 2.54 | .139 | .19 |
|  | Time | .49 | .498 | .04 | 60.85 | .000 | .85 | 5.94 | .033 | .35 |
|  | Drug × Time | .25 | .627 | .02 | 5.16 | .044 | .32 | 2.73 | .126 | .20 |
| SubC-LatV | Drug | .36 | .561 | .03 | 13.17 | .004 | .54 | 2.95 | .114 | .21 |
|  | Time | 1.16 | .305 | .10 | 64.11 | .000 | .85 | 7.39 | .020 | .40 |
|  | Drug × Time | .05 | .824 | .00 | 1.30 | .278 | .11 | 3.14 | .104 | .22 |
| SM-MedV | Drug | .83 | .381 | .07 | 15.83 | .002 | .59 | 2.34 | .154 | .18 |
|  | Time | .12 | .731 | .01 | 85.09 | .000 | .89 | 10.20 | .009 | .48 |
|  | Drug × Time | .70 | .421 | .06 | 1.37 | .267 | .11 | .76 | .403 | .06 |
| SM-OccP | Drug | 1.14 | .309 | .09 | 6.28 | .029 | .36 | 2.28 | .159 | .17 |
|  | Time | .21 | .654 | .02 | 56.45 | .000 | .84 | 7.59 | .019 | .41 |
|  | Drug × Time | 1.57 | .236 | .13 | 4.39 | .060 | .29 | 1.67 | .222 | .13 |
| SM-LatV | Drug | .11 | .741 | .01 | 13.19 | .004 | .55 | 3.67 | .082 | .25 |
|  | Time | .17 | .685 | .02 | 60.31 | .000 | .85 | 10.34 | .008 | .48 |
|  | Drug × Time | .63 | .444 | .05 | 2.74 | .126 | .20 | 2.52 | .141 | .19 |
| MedV-OccP | Drug | .09 | .765 | .01 | 8.32 | .015 | .43 | 2.67 | .131 | .20 |
|  | Time | 1.74 | .214 | .14 | 76.58 | .000 | .87 | 8.08 | .016 | .42 |
|  | Drug × Time | 4.06 | .069 | .27 | 1.28 | .281 | .10 | 1.13 | .311 | .09 |
| MedV-LatV | Drug | 8.14 | .016 | .43 | 14.39 | .003 | .57 | 1.91 | .195 | .15 |
|  | Time | 4.62 | .055 | .30 | 62.26 | .000 | .85 | 11.03 | .007 | .50 |
|  | Drug × Time | .76 | .402 | .06 | 1.22 | .293 | .10 | 3.76 | .079 | .25 |
| OccP-LatV | Drug | .59 | .459 | .05 | 6.50 | .027 | .37 | 3.18 | .102 | .22 |
|  | Time | 1.77 | .211 | .14 | 55.50 | .000 | .83 | 7.57 | .019 | .41 |
|  | Drug × Time | .12 | .738 | .01 | 3.41 | .092 | .24 | 4.45 | .059 | .29 |

*Motion Analyses*

Because head movement can be a major confound in fMRI studies, especially in pharmaco-fMRI studies, we compared head motion between drug conditions and between the first and second halves of scans. Framewise displacement (FD) was significantly greater in the first half of the salvinorin A scan compared to the first half of the placebo scan (*t*(11) = 2.23, *p* = .047), but this was not the case when comparing the second halves of scans (*t*(11) = .70, *p* > .250). Although FD was greater in the first compared to the second half of the salvinorin A scan (*t*(11) = 2.78, *p* = .018), there was also a similar trend in the placebo scan (*t*(11) = 1.97, *p* = .074)). Similarly, the number of scrubbed volumes (spikes) was greater in the first half of the salvinorin A scan (*M* = 70.08, *SD* = 51.01) compared to the first half of the placebo scan (*M* = 43.50, *SD* = 34.88; *t*(11) = 3.42, *p* = .006), and this was not significant when comparing the second halves (placebo: *M* = 13.17, *SD* = 13.71; salvinorin A: *M* = 26.58, *SD* = 33.66; *t*(11) = 1.69, *p* = .119). In both scans, there were more spikes in the first compared to the second halves (placebo: *t*(11) = 2.96, *p* = .013; salvinorin A: *t*(11) = 3.20, *p* = .008).

In order to explore the degree to which our effects may have been impacted by this increased motion during the first half of the salvinorin A scan, we assessed whether FD and spikes during the salvinorin A scan were correlated with subjective drug strength (area underneath the curve for the first 20 minutes from the practice session) and whether changes in motion parameters from the placebo scan to the salvinorin A scan were correlated with changes in sFC, dFC, and eFC from the placebo scan to the salvinorin A scan (using a liberal, uncorrected α of .05). Furthermore, we preprocessed our data using both 6 (displacements and rotations) and 24 (displacements and rotations, their derivatives, and squares of all of these) motion regressors to see if any observed effects were attenuated or were augmented as the number of motion regressors was increased. Finally, we looked at the relationship between physical distance between nodes (edge length in mm) and the correlation between edge strength and motion. When motion is high, longer range sFC edges can especially become stronger, resulting in larger correlations between motion and sFC in these long-range edges ^16^.

Motion during the salvinorin A scan was not correlated with subjective drug strength (FD: *r* = .17, *p* > .250; spikes: *r* = .07, *p* > .250), and spikes did not systematically correlate with any functional connectivity measure (all *p* > .05 uncorrected). Therefore, the discussion of correlations between motion and functional connectivity below is limited to correlations between FD and functional connectivity.

More rigorous motion correction did not drastically change the sFC findings (S6a and S6b), and the decrease in default mode network (DMN) sFC under salvinorin A during the first half of the scan was slightly larger with more rigorous motion correction. Only a few changes in sFC network interactions correlated with motion (Fig. S6c and S6d), and considering we used an uncorrected threshold of *p* < .05, it is possible these correlations were spurious. Of importance, the change in default mode network (DMN) sFC was not correlated with motion in either the more or less rigorous motion correction models. Moreover, correlations between FD and sFC present with more rigorous motion correction were not those present with less rigorous motion correction (and vice versa), suggesting that there may have been overcompensation with motion correction.


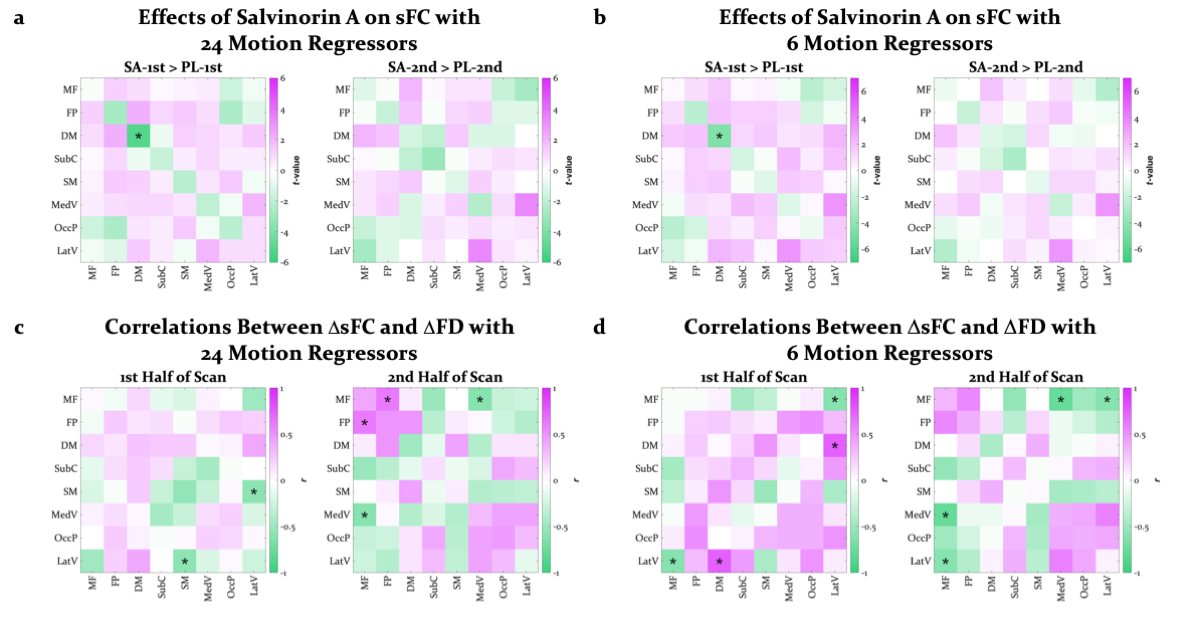


**Figure S6. (a)** and **(b)** Differences (*t*-values) in within- and between-network static functional connectivity (sFC) for salvinorin A (SA) vs. placebo (PL) in the first and second half of scans using 24 and 6 motion regressors. Each row and column represent a single brain network as defined by the Shen functional brain atlas. The diagonal and off-diagonal cells represent differences in within- and between-network connectivity, respectively. Panel **(a)** is the same as Fig. 2a in the main paper. **p* < .05, Holm-Bonferroni corrected for all 36 within- and between-network comparisons. **(c)** and **(d)** Correlations (Pearson’s *r*) across participants between these changes in connectivity and changes in motion (framewise displacement or FD) between the salvinorin A and placebo scans with 24 and 6 motion regressors. **p* < .05, uncorrected.

In contrast, with more rigorous motion correction, drug-induced decreases in dFC became larger (Fig. S7a and S7b) and drug-induced increases in eFC became smaller (Fig. S8a and S8b). In the case of dFC, decreases in dFC under salvinorin A were correlated with changes in FD for most network interactions with less motion correction, and about half of these correlations were no longer significant after more rigorous motion correction (Fig. S7c and S7d). These correlations were negative suggesting that the decreases in dFC under salvinorin A did in fact become larger with more motion. Conversely, drug-induced changes in eFC became more correlated with motion with more rigorous motion correction, again suggesting that there may have been overcompensation with more rigorous motion correction (Fig. S8c and S8d). These correlations were also negative, suggesting that the increases in eFC under salvinorin A were potentially attenuated with more motion, though this may have been due to the high correlation between eFC and motion in the first half of the placebo scan (see analysis below).


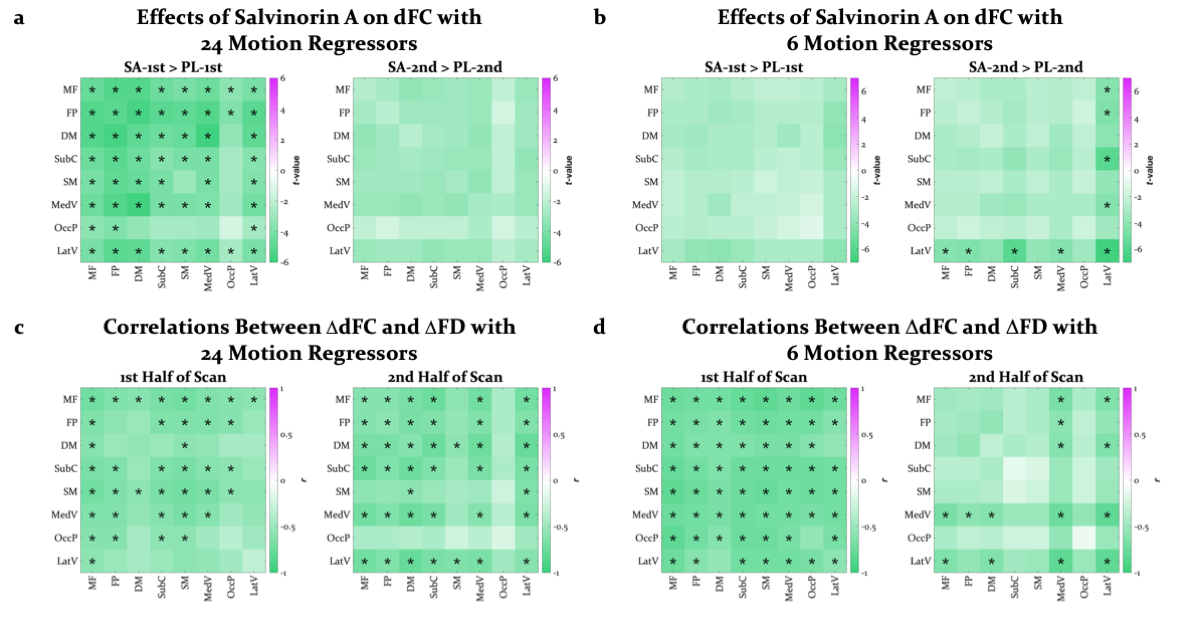


**Figure S7. (a)** and **(b)** Differences (*t*-values) in within- and between-network dynamic functional connectivity (dFC) for salvinorin A (SA) vs. placebo (PL) in the first and second half of scans using 24 and 6 motion regressors. Each row and column represent a single brain network as defined by the Shen functional brain atlas. The diagonal and off-diagonal cells represent differences in within- and between-network connectivity, respectively. Panel **(a)** is the same as Fig. 3b in the main paper. **p* < .05, Holm-Bonferroni corrected for all 36 within- and between-network comparisons. **(c)** and **(d)** Correlations (Pearson’s *r*) across participants between these changes in connectivity and changes in motion (framewise displacement or FD) between the salvinorin A and placebo scans with 24 and 6 motion regressors. **p* < .05, uncorrected.


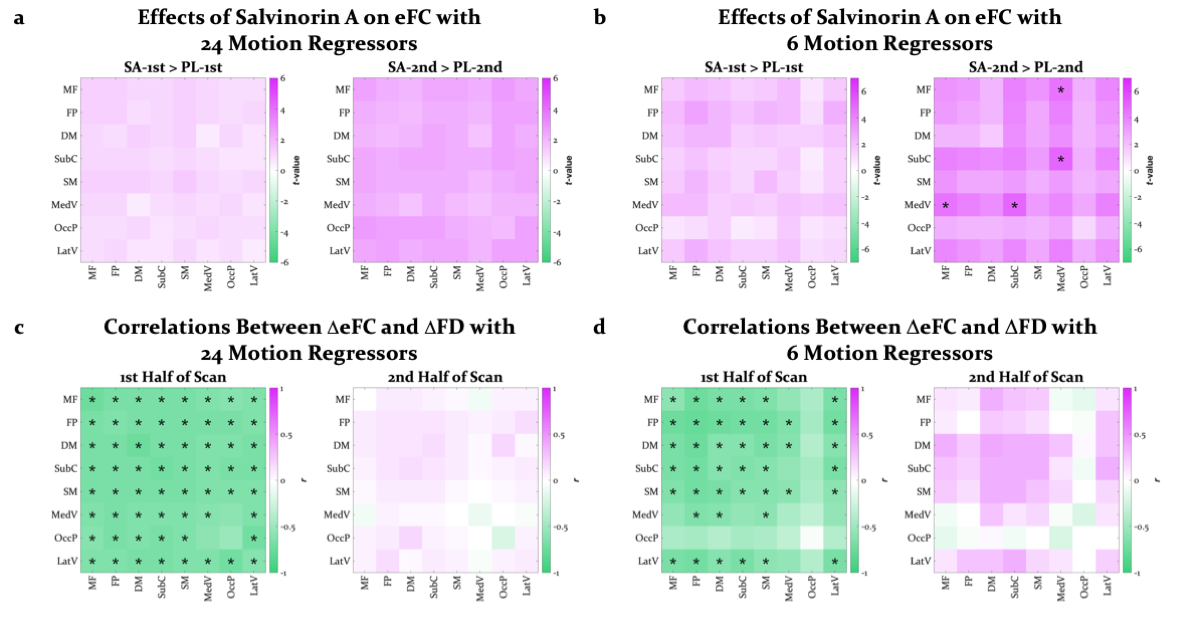


**Figure S8. (a)** and **(b)** Differences (*t*-values) in within- and between-network entropic functional connectivity (sFC) for salvinorin A (SA) vs. placebo (PL) in the first and second half of scans using 24 and 6 motion regressors. Each row and column represent a single brain network as defined by the Shen functional brain atlas. The diagonal and off-diagonal cells represent differences in within- and between-network connectivity, respectively. Panel **(a)** is the same as Fig. 3d in the main paper. **p* < .05, Holm-Bonferroni corrected for all 36 within- and between-network comparisons. **(c)** and **(d)** Correlations (Pearson’s *r*) across participants between these changes in connectivity and changes in motion (framewise displacement or FD) between the salvinorin A and placebo scans with 24 and 6 motion regressors. **p* < .05, uncorrected.

Finally, as can be seen in Fig. S9, no significant or substantial edgewise correlations between internodal distance and change in sFC of an edge between more or less rigorous motion correction models were observed (placebo first half: *r* = .17; placebo second half: *r* = .16; salvinorin A first half: *r* = .05; salvinorin A second half: *r* = -.06). This suggests that changes in sFC between drug conditions for long-distance edges are unlikely to have been influenced by motion. Distance between nodes was also not associated with dFC (placebo first half: *r* = .01; placebo second half: *r* = .04; salvinorin A first half: *r* = -.06; salvinorin A second half: *r* = .02) or eFC (placebo first half: *r* = .02; placebo second half: *r* = -.06; salvinorin A first half: *r* = .01; salvinorin A second half: *r* = .02). However, as can be seen in Fig. S11 and S12, on average, dFC and eFC were associated with motion, with correlations between motion and dFC particularly strong in salvinorin A conditions and correlations between motion and eFC particularly high and positive in the first half of the placebo condition. These motion-induced increases in eFC in the first half of the placebo condition coupled with the motion-induced decreases in eFC in the first half of the salvinorin condition may help explain why salvinorin A-induced increases in eFC were small.


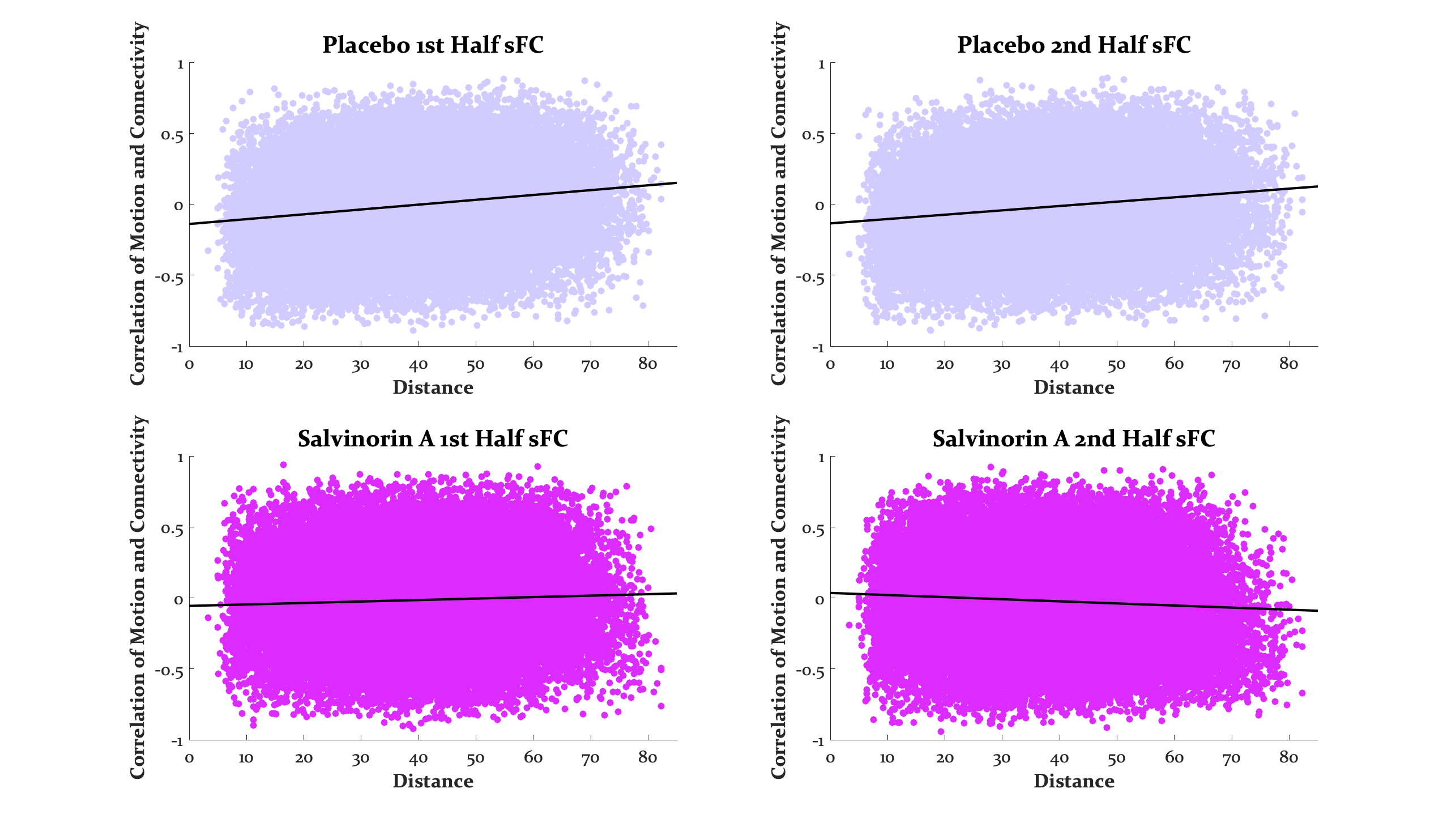


**Figure S9.** Relationship between distance between nodes and the modulation of motion and static functional connectivity (sFC). This relationship was small and, if anything, stronger in the placebo scan (i.e., the scan with less motion), suggesting that increases in functional connectivity between drug conditions was not related to motion.

**
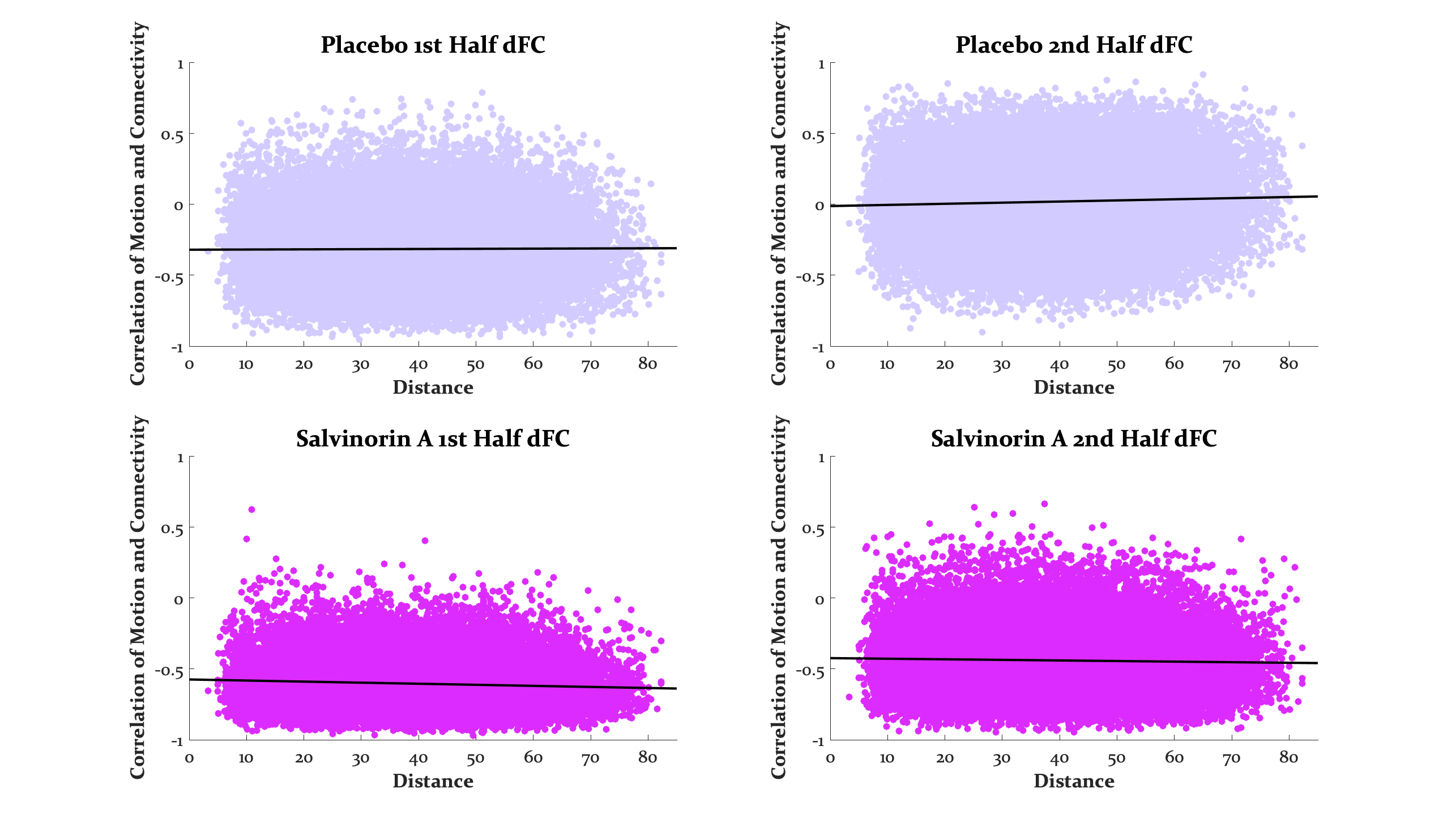
**

**Figure S10.** Relationship between distance between nodes and the modulation of motion and dynamic functional connectivity (dFC). Although the distance between nodes did not modulate this relationship, it can be seen that edgewise dFC was related to motion, especially in the salvinorin A condition.


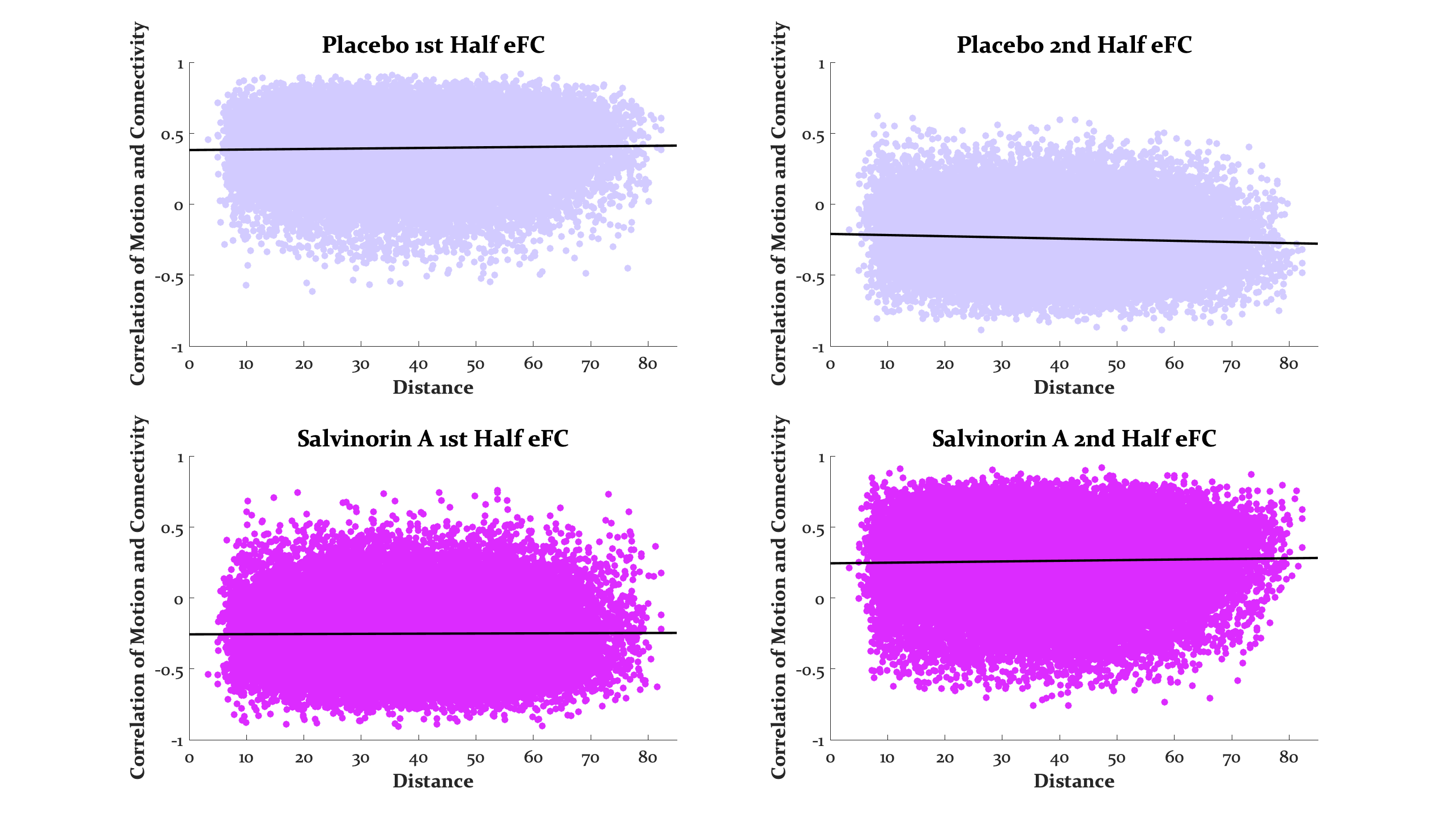


**Figures S11.** Relationship between distance between nodes and the modulation of motion and entropic functional connectivity (eFC). Although the distance between nodes did not modulate this relationship, it can be seen that edgewise eFC was related to motion, especially in the first half of the placebo scan.

*Partial Least Squares Discriminant Analyses Combining Across Connectomes*


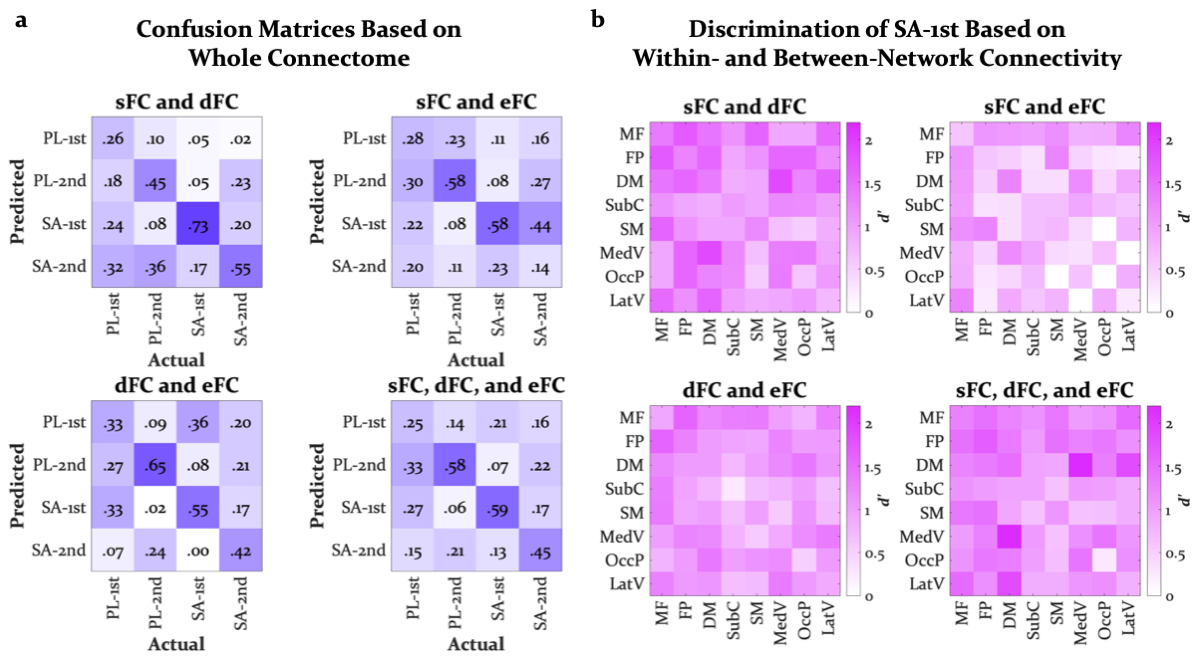


**Figure S12.** (**a**) Confusion matrices from the leave-one-participant-out cross-validation procedure, training partial least squares models on whole-brain combinations of static, dynamic, and entropic connectivity. (**b**) Discrimination (*d*’) of first half salvinorin A scans from the leave-one-participant-out cross-validation procedure, training partial least squares models on within- and between-network combinations of static, dynamic, and entropic connectivity. SA = salvinorin A, PL = placebo, sFC = static functional connectivity, dFC = dynamic functional connectivity, eFC = entropic functional connectivity, MF = medial frontal network, FP = frontoparietal network, DM = default mode network, SubC = subcortical-cerebellum network (includes the salience network), SM = somatosensory-motor network, MedV = medial visual network, OccP = occipital pole network, and LatV = lateral visual network.

**References**

1. Johnson, M. W., MacLean, K. A., Reissig, C. J., Prisinzano, T. E. & Griffiths, R. R. Human psychopharmacology and dose-effects of salvinorin A, a kappa opioid agonist hallucinogen present in the plant Salvia divinorum. *Drug and Alcohol Dependence* **115**, 150–155 (2011).

2. MacLean, K. A., Johnson, M. W., Reissig, C. J., Prisinzano, T. E. & Griffiths, R. R. Dose-related effects of salvinorin A in humans: dissociative, hallucinogenic, and memory effects. *Psychopharmacology* **226**, 381–392 (2013).

3. Calhoun, V. D. *et al.* The impact of T1 versus EPI spatial normalization templates for fMRI data analyses: Impact of T1 vs EPI Spatial Normalization Templates. *Human Brain Mapping* **38**, 5331–5342 (2017).

4. Behzadi, Y., Restom, K., Liau, J. & Liu, T. T. A component based noise correction method (CompCor) for BOLD and perfusion based fMRI. *NeuroImage* **37**, 90–101 (2007).

5. Satterthwaite, T. D. *et al.* An improved framework for confound regression and filtering for control of motion artifact in the preprocessing of resting-state functional connectivity data. *NeuroImage* **64**, 240–256 (2013).

6. Power, J. D., Barnes, K. A., Snyder, A. Z., Schlaggar, B. L. & Petersen, S. E. Spurious but systematic correlations in functional connectivity MRI networks arise from subject motion. *NeuroImage* **59**, 2142–2154 (2012).

7. Shen, X., Tokoglu, F., Papademetris, X. & Constable, R. T. Groupwise whole-brain parcellation from resting-state fMRI data for network node identification. *NeuroImage* **82**, 403–415 (2013).

8. Barrett, F. S., Johnson, M. W. & Griffiths, R. R. Validation of the revised Mystical Experience Questionnaire in experimental sessions with psilocybin. *Journal of Psychopharmacology* **29**, 1182–1190 (2015).

9. MacLean, K. A., Leoutsakos, J.-M. S., Johnson, M. W. & Griffiths, R. R. Factor Analysis of the Mystical Experience Questionnaire: A Study of Experiences Occasioned by the Hallucinogen Psilocybin. *Journal for the Scientific Study of Religion* **51**, 721–737 (2012).

10. Barrett, F. S., Bradstreet, M. P., Leoutsakos, J.-M. S., Johnson, M. W. & Griffiths, R. R. The Challenging Experience Questionnaire: Characterization of challenging experiences with psilocybin mushrooms. *Journal of Psychopharmacology* **30**, 1279–1295 (2016).

11. Mehling, W. E. *et al.* The Multidimensional Assessment of Interoceptive Awareness (MAIA). *PLoS ONE* **7**, e48230 (2012).

12. Maqueda, A. E. *et al.* Salvinorin-A Induces Intense Dissociative Effects, Blocking External Sensory Perception and Modulating Interoception and Sense of Body Ownership in Humans. *International Journal of Neuropsychopharmacology* **18**, pyv065 (2015).

13. Studerus, E., Gamma, A. & Vollenweider, F. X. Psychometric Evaluation of the Altered States of Consciousness Rating Scale (OAV). *PLoS ONE* **5**, e12412 (2010).

14. Burioka, N. *et al.* Approximate Entropy in the Electroencephalogram during Wake and Sleep. *Clinical EEG and Neuroscience* **36**, 21–24 (2005).

15. Hou, F. *et al.* Complexity of Wake Electroencephalography Correlates With Slow Wave Activity After Sleep Onset. *Frontiers in Neuroscience* **12**, (2018).

16. Satterthwaite, T. D. *et al.* Impact of in-scanner head motion on multiple measures of functional connectivity: Relevance for studies of neurodevelopment in youth. *NeuroImage* **60**, 623–632 (2012).
